# A systematic review of the research progress of non-coding RNA in neuroinflammation and immune regulation in cerebral infarction/ischemia-reperfusion injury

**DOI:** 10.3389/fimmu.2022.930171

**Published:** 2022-10-07

**Authors:** Kailin Yang, Liuting Zeng, Anqi Ge, Shanshan Wang, Jinsong Zeng, Xiao Yuan, Zhigang Mei, Guozuo Wang, Jinwen Ge

**Affiliations:** ^1^ Key Laboratory of Hunan Province for Integrated Traditional Chinese and Western Medicine on Prevention and Treatment of Cardio-Cerebral Diseases, Hunan University of Chinese Medicine, Changsha, China; ^2^ The First Hospital of Hunan University of Chinese Medicine, Changsha, China; ^3^ Hunan Academy of Chinese Medicine, Changsha, China

**Keywords:** ischemic stroke, non-coding RNA (lncRNA, miRNA, circRNA), neuroimmune inflammation, glial cells, epigenetic regulation

## Abstract

Cerebral infarction/ischemia-reperfusion injury is currently the disease with the highest mortality and disability rate of cardiovascular disease. Current studies have shown that nerve cells die of ischemia several hours after ischemic stroke, which activates the innate immune response in the brain, promotes the production of neurotoxic substances such as inflammatory cytokines, chemokines, reactive oxygen species and − nitrogen oxide, and mediates the destruction of blood-brain barrier and the occurrence of a series of inflammatory cascade reactions. Meanwhile, the expression of adhesion molecules in cerebral vascular endothelial cells increased, and immune inflammatory cells such as polymorphonuclear neutrophils, lymphocytes and mononuclear macrophages passed through vascular endothelial cells and entered the brain tissue. These cells recognize antigens exposed by the central nervous system in the brain, activate adaptive immune responses, and further mediate secondary neuronal damage, aggravating neurological deficits. In order to reduce the above-mentioned damage, the body induces peripheral immunosuppressive responses through negative feedback, which increases the incidence of post-stroke infection. This process is accompanied by changes in the immune status of the ischemic brain tissue in local and systemic systems. A growing number of studies implicate noncoding RNAs (ncRNAs) as novel epigenetic regulatory elements in the dysfunction of various cell subsets in the neurovascular unit after cerebral infarction/ischemia-reperfusion injury. In particular, recent studies have revealed advances in ncRNA biology that greatly expand the understanding of epigenetic regulation of immune responses and inflammation after cerebral infarction/ischemia-reperfusion injury. Identification of aberrant expression patterns and associated biological effects of ncRNAs in patients revealed their potential as novel biomarkers and therapeutic targets for cerebral infarction/ischemia-reperfusion injury. Therefore, this review systematically presents recent studies on the involvement of ncRNAs in cerebral infarction/ischemia-reperfusion injury and neuroimmune inflammatory cascades, and elucidates the functions and mechanisms of cerebral infarction/ischemia-reperfusion-related ncRNAs, providing new opportunities for the discovery of disease biomarkers and targeted therapy. Furthermore, this review introduces clustered regularly interspaced short palindromic repeats (CRISPR)-Display as a possible transformative tool for studying lncRNAs. In the future, ncRNA is expected to be used as a target for diagnosing cerebral infarction/ischemia-reperfusion injury, judging its prognosis and treatment, thereby significantly improving the prognosis of patients.

## 1 Introduction

Stroke is a disease characterized by a series of clinical neurological symptoms caused by hemorrhage of brain tissue or blood supply disorder, which is also known as stroke. Stroke has a high morbidity, mortality and disability rate, which brings a huge burden to patients and society (1). At present, stroke is the second leading cause of death in the world and the first leading cause of death in my country, and its incidence is still increasing (2, 3). Stroke is generally divided into ischemic and hemorrhagic stroke, and ischemic stroke accounts for about 87% (4). Numerous neurological damage activities, such as stress, hypoxia, inflammation, and cerebral edema, may be caused by cerebral ischemia, and may lead to neuronal apoptosis in the ischemic center of the brain (5, 6). The main treatment options for stroke include thrombolysis, anticoagulation, antihypertensive, plasmin reduction, and catheter intervention. Thrombolytic drugs and neuroprotective drugs are more commonly used (7–9). However, vascular recanalization after thrombolysis can lead to ischemia-reperfusion injury, and rt-PAs drugs have no effect on protecting or reversing neuronal ischemia-reperfusion injury (10, 11). Therefore, it has become the focus of current research to grasp the pathogenesis of stroke as a whole, find therapeutic targets, and provide new methods for ischemic stroke research at the level of molecular regulation mechanisms.

Neuroinflammation is an inflammatory process that occurs in the central nervous system and is an adaptive response to tissue damage/infection (12), which is a double-edged sword (13). The transient up-regulated inflammatory process at the beginning of ischemic stroke can play a certain protective role, but as the inflammatory stimulus continues to expand, a series of inflammatory cascades will occur, resulting in secondary brain tissue damage and poor functional recovery (14). Neuroinflammation involves all cells in the central system, but rapid activation of microglia is the first sign of neuroinflammation (15). Noncoding RNAs (ncRNAs) are key regulators of gene expression, and the most studied ncRNAs are long noncoding RNAs (lncRNAs), microRNAs (miRNAs) and circular RNAs (circRNAs) (16). For ischemic stroke, it is crucial to find biomarkers for early diagnosis and guidance of treatment (17). The changes of ncRNA expression profiles during the ischemic stroke process have gradually attracted everyone’s attention (18, 19). The relative stability, specificity, and reproducibility of ncRNA make it a promising biomarker for early identification of diseases (20). This review mainly focuses on ncRNAs related to neuroinflammation in cerebral infarction/ischemia-reperfusion injury, thereby providing new directions for the diagnosis or treatment of ischemic stroke.

## 2 Methods

### 2.1 Search strategy

The reviewers conducted a systematic and comprehensive search of the literature for articles discussing non-coding RNAs and cerebral infarction/cerebral ischemia-reperfusion injury. The database include Web of Science, Wanfang Database, Pubmed, China National Knowledge Infrastructure (CNKI), Sinomed, VIP Database, Medline Complete, Embase, ClinicalTrials.gov and Cochrane Library with keywords “non-coding RNA”, “lncRNA”, “miRNA”, “circRNA”, “cerebral infarction”, “cerebral ischemia-reperfusion injury”, etc. The retrieval time from inception to April 1st, 2022. Taking Pubmed and Embase as examples, the search strategy and all keywords was included in **Table S1**.

### 2.2 Inclusion and exclusion criteria

The inclusion criteria were: (1) Participants: It can be animals, patients or cells with cerebral infarction or cerebral ischemia-reperfusion injury, regardless of type, race, etc., and must comply with medical ethics. (2) Intervention: The type of intervention is not limited, but needs to comply with medical ethics. (3) Outcomes: It contains changes in any Non-coding RNA (lncRNA, miRNA, circRNA). (4) Study design: Basic or clinical trials.

The exclusion criteria were: (1) Research that does not conform to medical ethics; (2) Research that has been retracted.

### 2.3 Literature screening and data management

**Figure 1:**
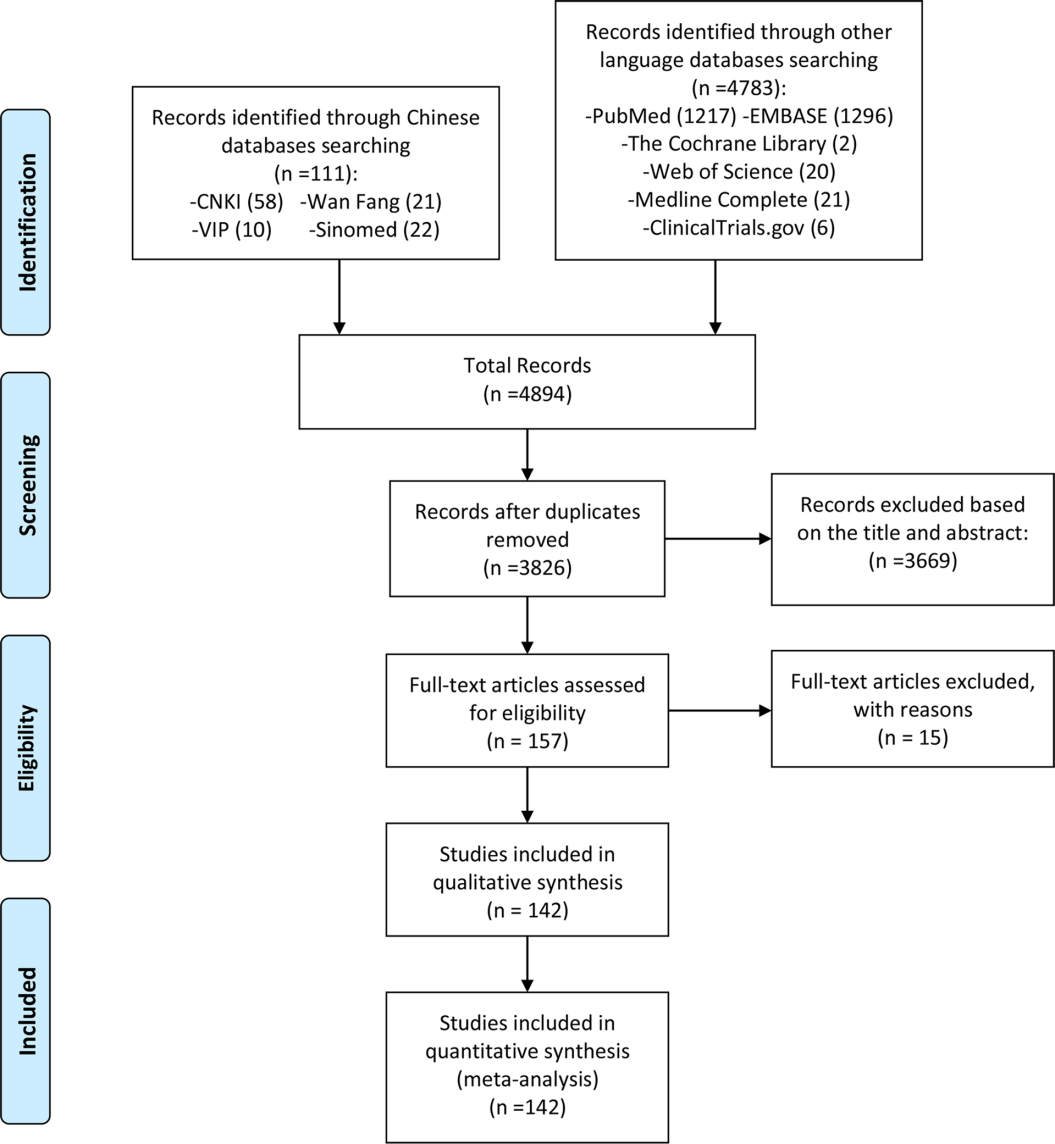
Flow diagram of literature screening.

The reviewers first conducted a preliminary search according to the search formula in **Table S1**, and the search results (4894 publications) were imported into the Endnote database. Duplicate publications were removed prior to further review. Two independent reviewers assessed articles for eligibility using the Liberal Acceleration Policy: Only one reviewer’s consent is required to advance a publication to the next stage of screening, but excluding a publication requires both reviewers’ consent. Two reviewers first assessed the article’s relevance to the review topic (ncRNA and cerebral infarction/cerebral ischemia-reperfusion injury) based on information in the title and abstract. Subsequently, two reviewers further screened the articles according to the inclusion and exclusion criteria, and included eligible articles (**Figure 1**). These included articles were shown in **Tables 1**–**6**.

**Table 1:** Summary of the mechanisms of lncRNAs in cerebral ischemia.

Author	LncRNA	Models/diseases	Species/cell source species	Targets	Pathway	Functions	Reference
Yin et al., 2019	SNHG12/LNC04080	MCAO, OGD/R	Mus musculus	miR-199a	Up-regulate SIRT1	SNHG12 targets miR-199a to upregulate SIRT1 expression, thereby attenuating cerebral ischemia/reperfusion injury through AMPK pathway activation.	(21)
Long et al., 2018	SNHG12/LNC04080	OGD/R	Mus musculus	miR-199a	Inhibit t-he expression of E-selectin, MCP 1, IL 6, and promote the expression of FGFb	SNHG12 inhibits OGD/R-induced endothelial cell injury, inflammatory response and angiogenesis by targeting miR-199a	(22)
Zhang et al., 2017b	MALAT1	MCAO, OGD/R	Rattus norvegicus	Not known	Promotes IL-6, MCP1 and E-selectin expression	Malat1 exerts anti-apoptotic and anti-inflammatory effects in the cerebral microvasculature to reduce ischemic cerebrovascular and parenchymal damage.	(23)
Zhang et al., 2018	MALAT1	MCAO, OGD/R	Mus musculus	miR-145	Involve in 15-LOX1/STAT3 signaling pathway	MALAT1 may regulate angiogenesis through the 15-LOX1/STAT3 signaling pathway, which may provide a key target for the treatment of hypoxic injury and a pathway for therapeutic angiogenesis.	(24)
Zhang et al., 2017b	MALAT1	MCAO, OGD/R	Rattus norvegicus	miR-375	Targeting PDE4D	Inhibition of MALAT1 attenuated CI/RI in rats by modulating the miR-375/PDE4D axis, inhibiting ROS levels, inflammatory factor levels, and apoptosis.	(23)
Wang et al., 2020a	MALAT1	MCAO, OGD/R	Mus musculus	miR-145	Inhibit AQP4	MALAT1 affects AQP4 expression by competitively binding to miR-145, thereby promoting cerebral ischemia-reperfusion injury.	(25)
Deng 2021	ANRIL	MCAO, OGD/R	Mus musculus	miR-671	Inhibit NF-κB signaling	Down-regulation of ANRIL reduces neuroinflammation by negatively regulating miR-671-5p to suppress NF-κB in IS models, providing a rationale for the protective effect of down-regulation of ANRIL in IS patients.	(26)
Feng et al., 2019	ANRIL	Acute ischemic stroke	Homo sapiens	Not known	Decrease CRP, TNF-α, IL-6, and promote IL-10	Downregulation of the circulating lncRNA ANRIL is associated with increased stroke risk, increased disease severity, and increased inflammation in patients with AIS.	(27)
Wang et al., 2017	H19	MCAO	Mus musculus	Not known	Reduce TNF-α and CD11b, increase Arg-1, CD206, IL-10	H19 promotes neuroinflammation by driving HDAC1-dependent M1 microglial polarization.	(28)
Wang et al., 2018b	NKILA	OGD/R	Mus musculus	miR-103 and miR-107	Inhibit NF-κB signaling	OGD/R induces upregulation of NKILA to inactivate NF-κB signaling, which mediates subsequent neuronal cell death.	(29)
Sun et al., 2021	SNHG14	OGD/R	Mus musculus	miR-30b-5p	Promote the expression of Atg5 and Beclin 1	SNHG14 promotes the expression of Atg5 and Beclin 1 by sponging miR-30b-5p, thereby activating autophagy and aggravating cerebral ischemia-reperfusion injury.	(30)
Qi et al., 2017	SNHG14	OGD/R	Mus musculus	miR-145-5p	Promote TNF-α and NO	Long non-coding RNA SNHG14 promotes microglial activation in cerebral infarction by regulating miR-145-5p/PLA2G4A	(31)
Zhang et al., 2021b	SNHG14	OGD/R	Mus musculus	miR-199b	Promote AQP4	Knockdown of SNHG14 attenuates ischemic brain injury by suppressing inflammation and oxidative stress through the miR-199b/AQP4 axis.	(32)
Zhong et al., 2019	SNHG14	OGD/R	Mus musculus	miR-136-5p	Promote ROCK1	SNHG14 promotes neural injury and inflammatory responses by increasing ROCK1 expression while reducing miR-136-5p levels in OGD/R-induced injury.	(33)
Sun et al., 2022	SNHG15	MCAO	Mus musculus	Not known	Promote the expression of p-STAT6	SNHG15 is a negative regulator of stroke-induced immunosuppressive inflammation.	(34)
Hu et al., 2021	SNHG15	OGD/R	Mus musculus	miR-302a-3p	Activiate STAT1/NF-κB pathway	Inhibition of SNHG15 ameliorated ischemia/hypoxia-induced neuronal damage and microglial inflammation by modulating the miR-302a-3p/STAT1/NF-κB pathway.	(35)
Zhang et al., 2020a	SNHG4	MCAO	Rattus norvegicus	miR-449c-5p	Up-regulate p-STAT6	SNHG4 regulates STAT6 and suppresses inflammation by adsorbing miR-449c-5p in microglia during cerebral ischemia-reperfusion injury.	(36)
Deng et al., 2019	Nespas	MCAO	Mus musculus	Not known	Inhibite NF-κB signaling	Nespas exerts anti-inflammatory and anti-apoptotic effects by inhibiting TRIM8-associated K63-linked polyubiquitination of TAK1.	(37)
Zhang and Zhang 2020	ZFASI	MCAO, OGD/R	Rattus norvegicus	miR-582-3p	Decrease Bax, Caspase-3, TNF-α, IL-1β, MCP-1, and increase Bcl-2	The lncRNA ZFAS1 protects neurons from injury and modulates inflammation, oxidative stress, apoptosis, cerebral ischemia-reperfusion injury by regulating miR-582-3p and NO levels.	(38)
Zhang et al., 2021c	MIAT	MCAO/R	Mus musculus	miR-874-3p	Decrease IL-1β	Lnc RNA MIAT impairs neural function in ischemic stroke by targeting IL1B by upregulating microRNA-874-3p	(39)
Wang et al., 2019	TUG1	OGD/R	Mus musculus	miR-145a-5p	Decrease TNF-α, IL-6, and increase IL-10; Inhibite NF-κB signaling	Lnc RNA TUG1 sponge Mir-145a-5p regulates microglial polarization	(40)
Liang et al., 2020	MEG3	MCAO, OGD/R	Rattus norvegicus	miR-485	Promotes the release of IL-1β and IL-18	The MEG3/miR-485/AIM2 axis promotes pyroptosis by activating caspase1 signaling during brain I/R.	(41)
Li et al., 2020a	MEG3	OGD/R	Mus musculus	Not known	Decrease CD86, iNOS, TNF-α, IL-1β, and increase CD206, Arg, IL-10, IL-4	Inhibition of MEG3 can alleviate cerebral ischemia-reperfusion injury by inhibiting M1 polarization and promoting M2 polarization through Krüppel-like factor 4 (KLF4)	(42)
Zhang et al., 2019a	1810034E14Rik	OGD/R	Mus musculus	Not known	Inhibite NF-κB signaling	Overexpression of 1810034E14Rik reduced the expression of inflammatory cytokines, and also inhibited the activation of microglia and inhibited the phosphorylation of p65.	(43)
Wang et al., 2020b	Gm15628	MCAO/R	Mus musculus	Not known	Increase IL-4	Silencing the LncRNA Maclpil in pro-inflammatory macrophages alleviates stroke via LCP1	(44)
Tian et al., 2021b	SNHG8	MCAO	Mus musculus	miR-425-5p	Inhibite NF-κB signaling, increase the expression of sirtuin1	The Snhg8/miR-425-5p/SIRT1/NF-κB axis plays a critical role in regulating cerebral ischemia-induced microglial inflammation and brain-blood barrier damage.	(45)
Zhang et al., 2021d	SNHG8	MCAO, OGD/R	Rattus norvegicus	miR-449c-5p	regulate SIRT1/FoxO1 signaling pathway	SNHG8 inhibits microglial activation and BBB permeability through the miR-449c-5p/SIRT1/FoxO1 pathway, thereby triggering protection against ischemic brain injury.	(46)
Jin 2021	NEAT1	MCAO	Mus musculus	Not known	Increase IL-1β, IL-6, TNF-α, and decrease IL-4, IL-10; activate JAK-STAT signaling pathway	Knockdown of Neat1 significantly attenuated brain injury by reducing the number of activated microglia and reducing their release of pro-inflammatory cytokines.	(47)
Han and Zhou 2019	NEAT1	OGD/R	Mus musculus	Not known	Regulate Wnt/β-catenin signaling	YY1-induced upregulation of NEAT1 promotes OGD/R injury and neuroinflammatory injury in microglia via the Wnt/β-catenin signaling pathway.	(48)
Zhang et al., 2021	NEAT1	OGD/R	Mus musculus	Not known	Activiate caspase-1	Gastrodin reduces cerebral ischemia/reperfusion injury by inhibiting pyroptosis by regulating the lncRNA NEAT1/miR-22-3p axis	(49)
Ni et al., 2020	NEAT1	OGD/R	Mus musculus	Not known	Inhibite AKT/STAT3 signaling pathway	The lncRNA NEAT1 may inhibit the polarization of microglia towards the M1 phenotype to reduce OGD/R-induced damage and reduce the activity of the AKT/STAT3 pathway.	(50)
Zheng et al., 2021	OIP5-AS1	ox-LDL-induced endothelial cell injury	Homo sapiens	miR-98-5p	Regulate TLR4/NF-κB signaling	LncRNA OIP5-AS1 accelerates ox-LDL-induced endothelial cell injury by regulating miR-98-5p-mediated HMGB1 through TLR4/NF-κB signaling pathway.	(51)
Zang et al., 2018	FIRRE	OGD/R	Mus musculus	Not known	Activate NF-κB signaling	FIRRE and NF-kB form a positive feedback loop to promote transcription of the NLRP3 inflammasome, leading to OGD/R injury in brain microglia.	(52)
Cheng et al., 2020	RMST	OGD/R	Mus musculus	miR-107	Activate p53/miR-107 signaling	LncRNA RMST-mediated transcription of miR-107 promotes OGD-induced neuronal apoptosis by interacting with hnRNPK	(53)
Sun et al., 2019	RMST	OGD/R	Mus musculus	Not known	Regulate TAK1-NF-κB	RMST competitively interacts with hnRNPK via the TAK1-mediated NF-κB pathway to promote OGD-induced M1 polarization in microglial cells.	(54)
Wang et al., 2021a	XIST	OGD/R	Mus musculus	miR-362	Regulate ROCK2	Knockout of XIST attenuates neuronal injury and inflammatory responses in cerebral ischemia/reperfusion injury by modulating the miR-362/ROCK2 axis	(55)
Wang et al., 2021b	XIST	MCAO	Mus musculus	miR-92a	Regulate Itgα5 and KLF4	Silencing of LncRNA XIST impairs angiogenesis after ischemic stroke and exacerbates cerebrovascular injury	(56)
Wen et al., 2017	Gm4419	OGD/R	Mus musculus	Not known	Increase TNF-α, IL-1β, IL-6; Activate NF-κB signaling.	LncRNA Gm4419 promotes OGD/R injury in brain microglia via IκB phosphorylation and NF-κB activation	(57)

**Table 2:** The summary of miRNAs changes in whole blood, serum, plasma, cerebrospinal fluid as diagnostic biomarkers in patients with stroke.

Authors	Sample source	Changes		Reference
		Downregulated	Up-regulated	
Jickling et al., 2014	Whole blood	miR-122, miR-148a, let-7i, miR-19a, miR-320d, miR-4429	miR-363, miR-487b	(58)
Sepramaniam et al., 2014	Whole blood	miR-182, miR-151-5p, miR-532-5p, miR-30c, miR-500*, miR-629, miR-7, miR-23a, miR-342-3p, miR-130a, miR-505*, miR-886-5p, miR-222, let-7d*, let-7c, miR-625, miR-502-5p, miR-574-3p, miR-335, miR-361-5p, miR-331-3p, miR-30e*, miR-208a, miR-22*, let-7a, miR-342-5p, miR-92a, miR-362-5p, miR-576-5p, miR-18a*, miR-106b*, miR-320b, let-7i, miR-652, miR-423-3p, let-7f, miR-183, miR-26b, miR-363, miR-186, miR-93*, miR-30b, let-7g, miR-502-3p, miR-1299, miR-96, miR-574-5p, let-7b*, miR-126, miR-192, miR-500, miR-23b, miR-324-5p, miR-320d, miR-20a, miR-340, miR-493*, miR-501-5p	miR-99a, miR-943, miR-933, miR-920, miR-675, miR-671-5p, miR-668, miR-659, miR-638, miR-637, miR-627, miR-623, miR-617, miR-611, miR-602, miR-585, miR-553, miR-552, miR-549, miR-525-5p, miR-498, miR-494, miR-490-3p, miR-488, miR-483-5p, miR-422a, miR-381, miR-370, miR-34b, miR-27a*, miR-26b*, miR-25*, miR-220c, miR-214, miR-210, miR-200b*, miR-198, miR-196a*, miR-187*, miR-184, miR-145, miR-135b, miR-1321, miR-129-5p, miR-1261, miR-125b-2*, let-7e	(59)
Chen et al., 2016	Plasma	Not reported	miR-144	(60)
Yang Li et al., 2016	Plasma	miR-223	miR-128b, miR-153 and miR-107	(61)
Mick et al., 2017	Plasma	Not reported	miR-320d, miR-656-3p, miR-3615, miR-124-3p, miR-941, miR-877-5p	(62)
Xiang et al., 2017	Plasma	Not reported	miR-15a, let-7i	(63)
Wang et al., 2018	Plasma	Not reported	miR-21-5p, miR-30a-5p	(64)
Jin and Xing 2017	Plasma	miR-19a, miR-296, miR-130a, miR-101, miR-378, miR-126	miR-185, miR-222, miR-206, miR-185, miR-221, miR-218	(65)
Wang et al., 2017	Serum	miR-221-3p, miR-382-5p	miR-221-3p	(66)
Chen et al., 2018	Serum	Not reported	miR-146b, miR-21	(67)
Ji et al., 2016	Serum	Not reported	miR-9 and -124	(68)
Li et al., 2015	Serum	Not reported	miR-1246, miR-532-5p, miR-32-3p, miR-106-5p	(69)
Chen et al., 2017	Serum	Not reported	miR-223	(70)
Wang et al., 2018	Serum	Not reported	miR-210	(71)
Vijayan et al., 2018	Serum	Not reported	PC-3p-57664, miR-211-5p, PC-5p-12969, miR-122-5p	(72)
Sørensen et al., 2017	CSF	Not reported	miR-9-5p, miR-128-3p, miR-9-3p, miR-124-3p	(73)
Peng et al., 2015	CSF	Not reported	let-7e	(74)

**Table 3:** Summary of miRNAs associated with prognosis.

Authors	Associated with good prognosis	Associated with poor prognosis	Reference
Tan et al., 2009	miR-320, miR-210	miR-103, miR-29	(75)
Griffiths-Jones et al., 2007	miR371-3p, miR-524, miR-520g, miR-1255A, miR-453, and miR-583	Not reported	(76)
Scherrer et al., 2017	Not reported	miR-150-5p	(77)
He et al., 2017	Not reported	miR-1203	(78)
Edwardson et al., 2018	Not reported	miR-941, miR-449b, miR-581	(79)

**Table 4:** The summary of the mechanisms of miRNAs in cerebral ischemia.

Author	miRNA	Models/diseases	Species/Cell source species	Pathway/target	Functions	Reference
Chen et al., 2021	miR-155	MCAO, OGD/R	Mus musculus	Regulate TLR4; decrease SOCS-1, Myd88; Activate NF-κB	MicroRNA-155 affects ischemic stroke cell injury through TLR4/MYD88 signaling pathway	(80)
Fan et al., 2018	miR-377	MCAO, OGD/R	Rattus norvegicus	Increase EGR2, VEGF	MiR-377 regulates inflammation and angiogenesis in rats after cerebral ischemic injury	(81)
Song et al., 2020	miR-1202	OGD/R	Homo sapiens	Inhibite TLR4/NF-κB signaling pathway	MiR-1202 exerts neuroprotective effect on OGD/R-induced HM cell inflammation by negatively regulating Rab1a involved in TLR4/NF-κB signaling pathway	(82)
Ge et al., 2016	miR-21-5p	In vitro scratch injury of microvascular endothelial cells	Rattus norvegicus	Regulate NF-κB signaling	miR-21-5p attenuates leakage of damaged brain microvascular endothelial barrier in vitro by inhibiting inflammation and apoptosis	(83)
Zhao et al., 2019	miR-20b	–	Homo sapiens	Regulate NLRP3 signaling pathway, increase IL-1β, IL-18	The miRNA-20b/NLRP3 axis may be a putative therapeutic target for cerebral ischemia.	(84)
Liu et al., 2015	miR-124	IS	Homo sapiens	Decrease CRP	Circulating miR-145 is associated with plasma hs-CRP in patients with acute ischemic stroke	(85)
Yang et al., 2015	miR-203	OGD/R	Mus musculus	Inhibite NF-κB signaling	miR-203 represents a novel target for regulating neuroinflammation and brain injury.	(86)
Tian et al., 2017	miR-93	MCAO/R	Mus musculus	Regulate Irak4 signaling	MiR-93 inhibits inflammation and apoptosis after cerebral ischemia-reperfusion by targeting IL-1 receptor-related kinase 4	(87)
Xie et al., 2017	miR-145-5p	MCAO/R	Rattus norvegicus	Regulate TNF-α	Interrupting miR-145-5p-Nurr1-TNF-α axis signaling in the acute phase may be an effective therapeutic strategy to alleviate neuronal injury during ischemia/reperfusion in rats.	(88)
Li et al., 2018b	miR-424	IS	Homo sapiens	Increase TNF-α, IGF-1	miR-424 prevents experimental stroke by inhibiting proliferation of microglia and activation of targeted cyclins.	(89)
Yin et al., 2017	miR-181c	–	Homo sapiens	Decrease TLR4	MiR-181c negatively regulates inflammatory responses in oxygen-glucose deprived microglia by targeting Toll-like receptor 4.	(90)
Mao et al., 2020	miR-195	OGD/R	Homo sapiens	Inhibite NF-κB signaling	MiR-195 attenuates oxygen-glucose deprivation/reperfusion-induced apoptosis by inhibiting IKKα-mediated NF-κB pathway	(91)
Xiang et al., 2019	miR-183	MCAO/R	Rattus norvegicus	Decrease IL-1β, IL-6 and TNF-α	miR-183 inhibits microglia activation and inflammatory factor expression in rats with cerebral ischemia-reperfusion through NF-κB signaling pathway	(92)
Wang 2018	miR-210	AIS	Homo sapiens	Increase IL-1β, IL-6, TNF-α, CCL2, CCL3	Inhibition of miR-210 suppresses pro-inflammatory responses and reduces acute brain injury from ischemic stroke in mice	(93)
Li et al. 2020b	miR-26b	MCAO/R	Mus musculus	Inhibite TLR signaling pathway	Exosome-carried miR-26b-5p regulates microglial M1 polarization after cerebral ischemia/reperfusion	(94)
Guo et al., 2021	miR-542-3p	OGD, MCAO	Mus musculus	Decrease TLR4	MSC-derived exosomal miR-542-3p prevents ischemia-induced glial inflammatory response by inhibiting TLR4	(95)
Dong et al., 2019	miR-22	MCAO	Rattus norvegicus	target p38 MAPK pathway	MiR-22 reduces inflammation in ischemic stroke via the p38 MAPK pathway	(96)
Zhang et al. 2021	miR-146a-5p	OGD, MCAO	Mus musculus	inhibit IRAK1/TRAF6 pathway	miR-146a-5p derived from hUMSC exosomes attenuates microglia-mediated neuroinflammation and neurological deficits after ischemic stroke	(97)
Huang et al., 2018	miR-210	MCAO	Mus musculus	Increase TNF-α, IL-1β, IL-6, CCL2 and CCL3	Inhibition of miR-210 suppresses pro-inflammatory responses and reduces brain damage in the acute phase of ischemic stroke	(98)
Li et al. 2020	miR-381-3p	MCAO	Mus musculus	Inhibite Cebpb and Map3k8	miR-381-3p downregulates Map3k8 and Cebpb to prevent ischemic stroke	(99)
Zheng et al., 2020	miR-421-3p	OGD, MCAO	Mus musculus	Target m6A Reader YTHDF	Prevention of inflammatory responses in cerebral ischemia/reperfusion injury by targeting m6A Reader YTHDF1 to inhibit p65 mRNA translation	(100)
Zhou et al., 2021	miR-19a/b-3p	OGD, MCAO	Rattus norvegicus	Increase FoxO3, SPHK1, NF-κB p65, and reduce SIRT1	miR-19a/b-3p promotes inflammatory responses during ischemia-reperfusion by targeting the SIRT1/FoxO3/SPHK1 axis.	(101)
Zhang et al., 2020	miR-665-3p	OGD	Mus musculus	Target TRIM8 to inhibit NF-κB signaling	miR-665-3p upregulation protects microglia from OGD-induced apoptosis and inflammatory responses by targeting TRIM8 to inhibit NF-κB signaling	(102)
Huang et al., 2022	miR-135b-5p	MCAO	Rattus norvegicus	Relieve neurological deficit and focal cerebral ischemia-reperfusion neuronal injury	Upregulation of miR-135b-5p reduces neuronal damage and inflammatory responses in PSCI by targeting NR3C2	(103)
Bao et al., 2021	miR-5787	OGD	THP-1 cell line (from Homo sapiens)	Inhibite LPS/TLR4	MiR-5787 attenuates macrophage-mediated inflammation in ischemic cerebral infarction by targeting TLR4/NF-κB	(104)
Liu et al., 2021	miR-200b-5p	MCAO	Mus musculus	Target SIRT1	Inhibition of miR-200b-5p improves neural function, reduces inflammation and neuronal apoptosis in MCAO mice	(105)
Shi et al., 2021	miR-532-5p	OGD, MCAO	Rattus norvegicus	Target CXCL1	MicroRNA-532-5p prevents cerebral ischemia-reperfusion injury by directly targeting CXCL1	(106)
Ye et al., 2021	miR-27-3p	MCAO	Rattus norvegicus	Target PPARγ	Serum exosomal microRNA-27-3p aggravates brain injury and inflammation in patients with acute cerebral infarction by targeting PPARγ	(107)
Tu et al., 2021	miR-34c-5p	OGD, MCAO	Rattus norvegicus	Regulates NCOA1 expression to inhibit nuclear factor-κB (NF-κB) activity, inhibits Caspase-3 and Bax, and up-regulates Bcl-2	miR-34c-5p may play an important role in cerebral ischemia/reperfusion injury through inflammatory and apoptotic signaling pathways	(108)
Shan et al., 2021	miR-221	MCAO	Mus musculus	Decreased expression of TNF-α, MCP-1, VCAM-1 and IL-6 CCL2 and CCL3	miR-221 exerts neuroprotective effects in ischemic stroke by inhibiting pro-inflammatory responses	(109)
Xie et al., 2020	miR-874-3p	MCAO	Mus musculus	Decrease CXCL12, TNF-α, IL-1, IL-6, IL-8, and increase IL-10	Up-regulation of microRNA-874-3p inhibits CXCL12 expression to promote angiogenesis and suppress inflammatory response in ischemic stroke	(110)
Deng et al., 2021	miR-671-5p	OGD, MCAO	Mus musculus	Inhibit NF-κB expression	miR-671-5p may be a viable therapeutic target for reducing neuroinflammation in patients	(26)
Zha et al., 2021	miR-153-5p	OGD, MCAO	Mus musculus	Targeting TLR4/p65/IkBa signaling pathway	Remote ischemic preconditioning partially reduces infarct volume and improves neurological function in acute ischemic stroke via miR-153-5p/TLR4/p65/IkBa signaling pathway	(111)
Liu et al., 2020	miR-92b-3p	OGD, MCAO	Rattus norvegicus	Targeting TRAF3 in PC12 cells	miR-92b-3p attenuated OGD/R-induced apoptosis, mitochondrial dysfunction, and inflammatory responses by targeting TRAF3.	(112)
Yang et al., 2017	miR-17-5p	MCAO	Mus musculus	Increase IL-6, MCP-1, VCAM1, TNF-α, decrease Bcl-2 and Bcl-w	Pharmacological inhibition of the miR-15a/16-1 cluster attenuates ischemic brain injury by upregulating anti-apoptotic proteins and inhibiting pro-inflammatory molecules.	(113)
Gamdzyk et al., 2020	miR-17-5p	OGD, MCAO	Rattus norvegicus	Inhibit TXNIP/NLRP3	PPAR-β/δ receptor agonist GW0742 activates miR-17-5p and inhibits TXNIP/NLRP3-mediated inflammation after hypoxic-ischemic injury in rat and PC12 cells	(114)
Tian et al., 2018	miR-216a	OGD, MCAO	Mus musculus	Inhibits JAK2/STAT3	Upregulation of miR-216a targeting JAK2 induces neuroprotection against ischemic injury in vitro and in vivo	(115)
Wang et al., 2018	miR-182-5p	MCAO	Rattus norvegicus	Regulate TLR4	MicroRNA-182-5p attenuates cerebral ischemia-reperfusion injury by targeting TLR4	(116)
Zhao et al., 2020	miR-223-3p	OGD, MCAO	Rattus norvegicus	Inhibit the expression of pro-inflammatory factors and promote the secretion of anti-inflammatory factors	Exosomes from MSCs overexpressing microRNA-223-3p attenuate cerebral ischemia by inhibiting microglial M1 polarization-mediated inflammation	(117)
Yang et al., 2021	miR-582-5p	OGD/R	PC12 cell line (from Rattus norvegicus)	Regulates cell viability, apoptosis, inflammation and oxidative stress	MiR-582-5p attenuates neonatal hypoxic-ischemic encephalopathy by targeting HMGB1 by inhibiting neuroinflammation and oxidative stress	(118)
Shi et al., 2020	miR-217	OGD, MCAO	Rattus norvegicus	Added IL-10	MEF2D-HDAC5/ND6 signaling pathway regulated by miR-217 is involved in oxidative stress and inflammatory response after cerebral ischemia	(119)
Chen et al., 2020	miR-193b-3p	OGD, MCAO	Rattus norvegicus	Regulates Leukotriene B4, Cysteinyl Leukotrienes and 5-LOX	MicroRNA-193b-3p attenuates focal cerebral ischemia-reperfusion injury in rats by inhibiting the expression of 5-lipoxygenase	(120)
Zhou et al., 2020	miR-199a	MCAO	Rattus norvegicus	promote inflammation	MiR-199a regulates autophagy and inflammation in rats with cerebral infarction by regulating mTOR expression	(121)
Fang et al., 2018	miR-544	OGD, MCAO	Mus musculus	Downregulation of IRAK4 expression	MicroRNA-544 inhibits inflammatory response and apoptosis after cerebral ischemia-reperfusion by targeting IRAK4	(122)
Yu et al., 2020	miR-199a-5p	OGD, MCAO	Rattus norvegicus	Inhibition of endoplasmic reticulum stress attenuates neuronal apoptosis and inflammation	HUVEC-derived miR-199a-5p inhibits ER stress-induced apoptosis and inflammation by targeting BIP.	(123)
Ni et al., 2015	let-7c-5p	OGD	HUVECs (from Homo sapiens)	Inhibit microglial activation	The inhibition of microglial activation by let-7c-5p overexpression may be related to the protective effect of ischemic injury.	(124)
Song et al., 2020	miR-1202	MCAO, OGD/R	Mus musculus	Inhibit TLR4/NF-κB inflammatory signaling pathway	MiR-1202 exerts neuroprotective effects by negatively regulating its target protein Rab1a	(82)
Liu et al., 2022	miR-665	MCAO, OGD/R	Mus musculus	Targeting ROCK2	MiR-665 participates in the protective effect of dexmedetomidine on ischemic stroke through ROCK2/NF-κB axis	(125)
Wang et al., 2020	miR-139	OGD/R	Human neuroblastoma cells SH-SY5Y (from Homo sapiens)	Inhibits pyroptosis and up-regulation of NLRP3, caspase-1, ASC, GSDMD Nterm, and release of IL-1β, IL-18 and LDH	Up-regulation of miR-139 exerts neuroprotection against OGD/R-induced nerve injury by negatively regulating c-Jun/NLRP3 inflammasome signaling.	(126)
Dong et al., 2016	miR-1202	OGD/R	Human microglial cells (from Homo sapiens)	Inhibit TLR4/NF-κB pathway	MiR-1202 exerts neuroprotective effect on OGD/R-induced HM cell inflammation by negatively regulating Rab1a involved in TLR4/NF-κB signaling pathway	(127)
Wu et al. 2020	miR-124-5p	MCAO, OGD/R	Rattus norvegicus	Modulation of NOX2 significantly inhibits NF-κB signaling activation and production of TNFα and IL-6	miR-124-5p/NOX2 Axis regulates ROS production and inflammatory microenvironment to prevent brain I/R injury	(128)
Ma et al., 2020	miR-155	MCAO	Mus musculus	Promotes M2 polarization of microglia	Resveratrol promotes M2 polarization of microglia and reduces neuroinflammation after cerebral ischemia by inhibiting miR-155	(129)
Li et al., 2019	miR-542-3p	MCAO	Mus musculus	Attenuated apoptosis, reactive oxygen species, and activation of inflammatory responses.	Adipose-derived mesenchymal stem cells alleviate ischemic brain injury in rats by regulating miR-21-3p/MAT2B signaling	(130)
Liu et al., 2016	miR-9	MCAO	Rattus norvegicus	Downregulate MCPIP1	Electroacupuncture inhibits inflammatory injury after ischemic stroke by targeting miR-9-mediated NF-κB signaling pathway	(131)
Song et al., 2022	miR-203-3p	MCAO	Rattus norvegicus	Inhibits neuronal apoptosis and inflammation	Sevoflurane protects mice from cerebral ischemic injury by modulating the microRNA-203-3p/HDAC4/Bcl-2 axis	(132)
Song et al., 2019	miR-181c-3p	MCAO	Rattus norvegicus	Inhibit CXCL1 expression	Cortical neuron-derived exosomal microRNA-181c-3p inhibits neuroinflammation by downregulating CXCL1 in astrocytes in a rat model of ischemic brain injury	(133)

**Table 5:** Summary of the mechanisms of circRNAs in cerebral ischemia.

Author	circRNA	Potential signal axis	Species	Model/disease	Function	Reference
Han et al., 2018	CircHECTD1 was up-regulated	–	Mus musculus and Homo sapiens	tMCAO mice and patient with AIS	Inhibition of Circhetd1 expression significantly reduces infarct size, reduces neuronal damage, and improves astrocyte activation	(134)
Wu et al., 2019	CircTLK1 was up-regulated	–	Mus musculus and Homo sapiens	MCAO mice and patient with AIS	Knockdown of CircTLK1 significantly reduces infarct volume, reduces neuronal damage and ameliorates nerve damage	(135)
Bai et al., 2018	CircDLGAP4 was down-regulation	CircDLGAP4/microRNA-143/E6-AP C-terminal domain E3 ubiquitin-protein ligase axis	Mus musculus and Homo sapiens	MCAO mice and patient with AIS	Up-regulation of circDLGAP4 expression significantly reduces nerve injury, infarct size, and blood-brain barrier damage	(136)
Li et al. 2020	Circ-0072309 was down-regulation	Circ-0072309/miR-100/mTOR axis	Mus musculus and Homo sapiens	Patient with IS and MCAO mice	Regulates cell survival and apoptosis	(137)
Tang et al. 2020	Circ_016719 was up-regulated	Circ_016719/miR-29c/MAP2k6 axis	Mus musculus	tMCAO	45Circ_016719 gene knockout significantly increases cell proliferation, down-regulates apoptosis, and significantly inhibits autophagy	(138)
Wu et al. 2020	CircCCDC9 was down-regulation	CircCCDC9/Notch1 signaling pathway	Mus musculus	tMCAO	Overexpressed circCCDC9 protects blood-brain barrier and inhibits apoptosis	(139)
Yang et al., 2020	CircMH 1 was down-regulation	CircSCMH1/MeCP2 axis	Mus musculus	Patients with AIS and Photothrombotic stroke in mice	Circscmh1 enhances neuronal plasticity and inhibits glial activation and peripheral immune cell infiltration	(140)
Wang et al., 2020	Si-CircHIPK2-NSC	–	Mus musculus	tMCAO	Si-Circhipk2 NSCs increase IS neuronal plasticity, provide durable neuroprotection, and significantly reduce functional deficits	(141)
Dai et al., 2021	Circ-HECTD1 was up-regulated	Circ-HECTD1/miR-133b/TRAF3 axis	Mus musculus	OGD and MCAO	Circ-hectd1 knockdown reduces OGD-induced neuronal death and infarct volume in vitro	(142)
Chen et al., 2020	CircSHOC2 was up-regulated	CircSHOC2/miR-7670-3p/SIRT1 axis	Mus musculus	OGD	Circshoc2 inhibits neuronal apoptosis and alleviates neuronal damage	(143)
Silva et al., 2020	hsa-Circ 0078299 and FXN	–	Homo sapiens	Patients with AIS	Neuroprotection and Brain Recovery	(144)
Zhu et al., 2019	PBMC-circ-DLGAP4 was up-regulated	–	Homo sapiens	Patients with AIS	Circ-DLGAP4 inversely correlates with inflammation and miR-143 expression in AIS patients	(145)
Peng et al., 2019	CircRNA HECTD1 was up-regulated	–	Homo sapiens	Patients with AIS	CircRNA HECTD1 expression is associated with higher disease risk, disease severity, inflammation and relapse	(146)
Bazan et al., 2017	CircR-284	–	Homo sapiens	24 asymptomatic and 17 symptomatic patients undergoing carotid endarterectomy	Serum Circ-284:MIR-221 can be used as an early warning factor for carotid plaque rupture and stroke	(147)
Zuo et al., 2020	CircFUNDC1	–	Homo sapiens	Patients with AIS	CircFUNDC1 can be used to build risk prediction models to predict SAI	(148)
Jiang et al., 2021	circleGLIS3	cGLIS3/miR-203/EIF4A3 axis	Mus musculus and Homo sapiens	MCAO mice and patient with AIS	cGLIS3 is a common regulator and diagnostic marker of brain neurodegeneration	(149)

**Table 6:** Summary of the mechanisms of circRNAs in cerebral ischemia (Sequencing results).

Authors	Changes in expression profiles	Specific core circRNA	Model/disease	Species	Potential biological processes and functions	Potential pathway/target gene	Reference
Mehta et al., 2017	1320 circRNAs differentially expressed (283 cases changed at least 1 reperfusion time point, 16 cases had 3 reperfusion time points change)	Circ_008018, Circ_015350, Circ_016128 were up-regulated, Circ_011137, Circ_001729, Circ_006696 were down-regulated	tMCAO	Mus musculus	Biological regulation, metabolic processes, cellular communication, and binding to proteins, ions, and nucleic acids	Mitogen-activated protein kinase signaling, cell cycle, actin cytoskeleton regulation and local adhesion	(150)
Liu et al., 2017	914 circRNAs were up-regulated and 113 circRNAs were down-regulated	mmu_CircRNA_40001, mmu_CircRNA_013120, mmu_CircRNA_4080	tMCAO	Mus musculus	Multiple biological processes, cell signaling pathways, and protein activities	Rap1 signaling channel and Hippo pathway	(151)
Ostolaza et al., 2020	Atherosclerosis versus thrombotic stroke: differential expression. Atherosclerosis vs. undetermined stroke: 87 up-regulated, 139 down-regulated. Thrombotic stroke versus undiagnosed stroke patients: 8 up-regulated, 9 down-regulated	HSA_CircRNA_102488	AIS	Homo sapiens	Fatty acid biosynthesis, lysine degradation, arrhythmogenic right ventricular cardiomyopathy or hypertrophic cardiomyopathy	The RBP site of hsa_CircRNA_102488 is clustered around AGO2 and FUS proteins	(152)
Lu et al., 2020	128,198,789 circRNA changes at 5 minutes, 3 hours, and 24 hours after IS	CircBBS2, CircPHKA2	AIS patients and tMCAO mice	Mus musculus and Homo sapiens	Immune processes, metabolic processes and bioadhesion	Hippo signaling pathway, extracellular matrix-receptor interaction and fatty acid metabolism	(153)
Li et al., 2020	2659 circRNAs were differentially expressed (73 circRNAs were significantly changed at 7 and 14 days after stroke)	mmu_Circ:chr2:74568941-74573626, mmu_Circ:chr8:8639206-8639489, mmu_Circ:chr18:14633543-14636618	MCAO	Mus musculus	Closely related to inflammation and self-repair, plays an important role in secondary thalamic neurodegeneration and remodeling after focal cortical infarction	Metabolic pathway, cancer pathway, PI3K-Akt signaling pathway, endocytosis, etc.	(154)
Dong et al., 2020	373 circRNAs were up-regulated and 148 circRNAs were down-regulated	hsa_cic:chr1:95609447-95616975, hsa_cic:chr15:55640530-55640923S were up-regulated; hsa_cic:chr9:80869752-80879232 were down-regulated	AIS	Homo sapiens	Neuroinflammation and Neuroimmunity	Metabolic pathways, mitogen-activated protein kinase signaling pathways, some inflammatory pathways, immune pathways, differentiation and apoptosis-related signaling pathways	(155)
Lin et al., 2016	3 circRNAs were up-regulated and 12 circRNAs were down-regulated	mmu-CircRNA-015947	OGD/R	Mus musculus	–	Apoptosis, metabolism and immune-related pathways	(156)
Duan et al., 2019	40 circRNAs were up-regulated and 47 circRNAs were down-regulated	CircRNA.17737, CircRNA.8828, CircRNA.14479 were up-regulated; CircRNA.1059, CircRNA.9967, CircRNA.6952 were down-regulated	MCAO	Rattus norvegicus	Nervous system development and endocytosis	cytoplasmic vesicles, vesicles, synapses, cytoskeleton, cytoplasmic vesicles	(157)
Li et al., 2020	659 circRNAs were up-regulated and 1611 circRNAs were down-regulated	hsa_Circ_0005548 was up-regulated; hsa_Circ_0000607 and hsa_Circ_0002465 were down-regulated	AIS	Homo sapiens	–	Endocytosis, energy metabolism, apoptosis, FOXO signaling pathway, platelet activation, neurotrophic factor signaling pathway, VEGF signaling pathway	(158)
Li et al., 2021	182 circRNAs were up-regulated and 176 circRNAs were down-regulated	hsa_CircRNA_0001599	Left atrial appendage stroke	Homo sapiens	–	Chromatin modification, autophagy, platelet activation, and neural precursor cell proliferation	(159)

## 3 Neuroimmune inflammatory mechanism of cerebral infarction/ischemia-reperfusion injury

Neuroimmune inflammation is an important component of the pathophysiology of ischemic stroke and a major target for the development of new therapeutics. Microglia, the first immune cells to sense ischemic stroke, are innate immune cells in the brain that express a variety of receptors involved in immune regulation and can recognize dead cells, pathogens, self-antigens, and neurotransmitters (160, 161). Microglia are quite sensitive to ischemia, and 12 hours after ischemic stroke, Cluster of Differentiation 11b (CD11b)-positive microglia in the infarcted area begin to fragment. After 24 hours, the number of microglia in the infarcted area was significantly reduced (162, 163), and the microglia in the ischemic penumbra were activated (164), with high expression of CD11b, CD45 and lonized calcium binding adapter molecule I (lba1). Peri-infarct microglial activation persists for weeks after ischemic stroke (162, 164, 165). Notably, microglia surrounding infarcts exhibit distinct pro- and anti-inflammatory phenotypes (164, 166, 167). However, in experimental ischemic stroke models, microglia do not appear to exhibit the typical MI and M2 phenotypes (168). In later stages, microglia aid in stroke recovery by phagocytosing dead cells and debris (169). However, microglia can also phagocytose surviving ischemic neurons that transiently express “eat me” signals, possibly increasing neuronal cell death around the infarct (170). Furthermore, infiltrating leukocytes, mainly including neutrophils and monocytes/macrophages, play a complex role in ischemic stroke. Neutrophil infiltration begins early in ischemic stroke (171) and attaches to endothelial cells by binding to various adhesion molecules (172). On the one hand, neutrophils exacerbate neuronal death by releasing neurotoxic proteases (173), and on the other hand, neutrophil infiltration aggravates blood flow obstruction, leading to the phenomenon of “no reflux” (174). Meanwhile, neutrophils damage the blood-brain barrier, induce hemorrhagic transformation after ischemic stroke, and aggravate neurological dysfunction (175). Monocyte recruitment in infarcts after ischemic stroke is regulated by adhesion molecules, chemokines and cytokines (176, 177). Monocyte/macrophage infiltration exacerbates brain damage in the acute phase of ischemic stroke, and monocytes/macrophages play an active role in the subacute phase after ischemic stroke, reducing the risk of hemorrhagic transformation (178, 179). Recent studies have shown that lymphocytes are closely related to ischemic stroke, and there is increasing evidence that T cell subsets are involved in the neuroimmune inflammatory function regulation impairment in the acute phase of ischemic stroke (180, 181). In summary, under the action of immune-inflammatory chemotactic cytokines and paracrine signals, various groups of immune cells regulate each other and jointly promote the pathological process of stroke in the early, middle and late stages. For example, after ischemic stroke, microglia are rapidly activated, and activated microglia release high levels of IL-23 1 day after ischemia-reperfusion and lead to infiltration of γδ T lymphocytes into the brain, thereby aggravating brain damage (182).

## 4 Mechanisms of lncRNAs involved in neuroinflammation and immune regulation in cerebral infarction/ischemia-reperfusion injury

**Figure 2:**
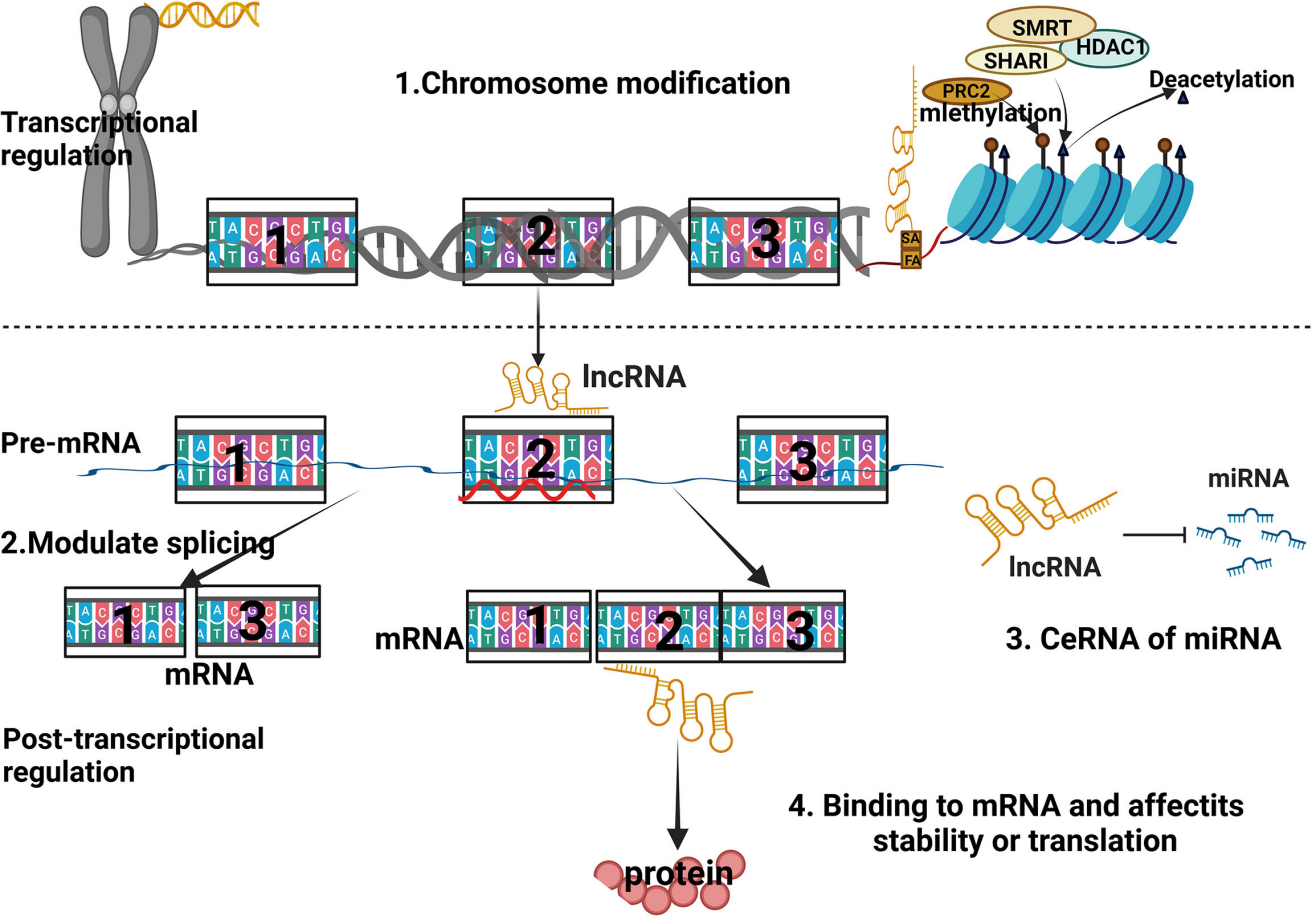
Schematic diagram of the regulatory mechanism of lncRNA.

LncRNAs are a class of transcripts over 200 nucleotides in length that do not encode proteins, but are involved in a variety of biological functions, such as epigenetic regulation, immune surveillance, and embryonic stem cell pluripotency (183–185). They are divided into 5 categories according to their position relative to protein-coding genes (186–188): (1) Sensory LncRNAs that overlap with coding mRNAs on the gene coding strand; (2) Antisense LncRNAs that overlap with coding mRNAs on the non-coding strand of the gene; (3) Bidirectional LncRNAs that share their transcription initiation sites with coding genes on opposite strands; (4) Intronic LncRNAs transcribed from the electronic region of the coding gene; (5) Intergenic LncRNAs located between coding genes. Their biological contributions are manifested in (189–191): (1) cis or trans transcriptional regulators; (2) mRNA processing, post-transcriptional control and protein activity regulators; (3) the organization of nuclear domains (**Figure 2**).

### 4.1 Mechanisms of SNHG12 involved in neuroinflammation and immune regulation in cerebral infarction/ischemia-reperfusion injury

Small nucleolar host gene 12 (SNHG12), also known as LNC04080, is about 1.8 kb long and is located on chromosome 1 p35. The lncRNA of the 3-region, comprising 1867 bases (192), encodes four small nucleolar RNAs (SNORA66, SNO-RA61, SNORA16A and SNORD99) through its spliced introns (193). SNHG12 acts as a competing endogenous RNA (ceRNA) by hiding multiple miRNA binding sites, regulating their downstream targets by “sponging” these miRNAs. Various LncRNA molecules, including lncRNAs, miRNAs, pseudogenes, and circular RNAs (circRNAs), share common miRNA response elements (MREs), thereby regulating each other through complex RNA networks and cellular processes (194). MREs exist in the 5’UTRs, coding sequences and 3’UTRs of genes, respectively (195, 196). LncRNA SNHG12 has been reported to have a higher density of MREs targeting miRNAs, thereby increasing the possibility of sharing and titrating miRNAs and preventing their binding to other transcripts (197).

SNHG12 was originally discovered in cancer cells and plays a key role in the proliferation and migration of cancer cells. Zhang et al. (2016) showed that 147 lncRNAs were up-regulated in brain microvascular endothelial cells after 16 h exposure to hypoxia and glucose (OGD), and SNHG12 was one of the most up-regulated lncRNAs (198). Up-regulated SNHG12 can reduce cell damage and induce autophagy, while knockdown of SNHG12 aggravates apoptosis and inflammation (199). Under OGD/R conditions, compared with the negative control group, Yin et al. (2019) found that SNHG12 upregulates SIRT1 by targeting miR-199a and activates the AMPK pathway to reverse the damage of miR-199a to brain microvascular endothelial cells and improved the inflammatory response and angiogenesis (21). Meanwhile, Zhao et al. (2018) reported that overexpression of SNHG12 improved the recovery of neurological function in the infarct border zone of MCAO mice, decreased infarct volume and miR-150 expression, and increased vascular density and VEGF expression. These suggest that the miR-199a/SIRT1/AMPK pathway and the miR-150/VEGF pathway play an important role in SNHG12 promoting ischemic stroke angiogenesis and regulating ischemic injury (200). Long et al. (2018) explored the role of SNHG12 during and after OGD/R injury through a series of cell *in vitro* experiments. They found that under OGD/R conditions, SNHG12 inhibited the expression of miR-199a, which in turn inhibited the death of brain microvascular endothelial cells (BMECs) and the expression of inflammatory cytokines E-selectin, MCP1 and IL6, and promoted the expression of angiogenic factors VEGFA and FGFb. In addition, SNHG12 also promoted the formation of capillary-like tubes after OGD/R. These results suggest that SNHG12 can inhibit the death and inflammatory response of BMECs under OGD/R conditions, promote BMEC angiogenesis after OGD/R injury, and improve the prognosis of ischemic stroke patients. Further exploration revealed that SNHG12 could directly target miR-199a, and that overexpression of miR-199a could improve BMEC death, inflammation, and angiogenesis (22). These studies suggest that SNHG12 has a protective effect on BMECs by inhibiting the pathophysiological process of miR-199a during and after OGD/R. This provides new clues for understanding the molecular mechanisms of microvascular injury, blood-brain barrier dysfunction and inflammation after ischemic stroke, and is of great significance for improving ischemic stroke treatment.

### 4.2 Mechanism of MALAT1 involved in stroke neuroinflammation

The gene encoding MALAT1 is located on human chromosome 11q13. This lncRNA exists in the nuclear plaque region of the nucleosome and is about 8.1 kb long. MALAT1 has a high expression abundance in various human tissues. In recent years, studies have shown that MALAT1 plays an important role in ischemic stroke in addition to being associated with a variety of cancers (201). Zhang et al. (2017b) pointed out that the expression of MALAT1 was increased in both OGD and MCAO models of cerebral microvascular endothelial cells. Silencing MALAT1 in rat brain microvessels increases the expression of pro-apoptotic factors such as cell death regulators, pro-inflammatory factors such as IL-6, monocyte chemoattractant protein 1 and E-selectin in brain microvascular endothelial cells (23). Compared with the normal control group, the infarct size of the brain tissue increased, the motor and sensory dysfunction aggravated, and the neurological function score decreased. This suggests that MALAT1 exerts a protective effect on cerebral vascular ischemia-hypoxic injury by inhibiting endothelial cell apoptosis and inflammatory response. MALAT1 is considered to be one of the most up-regulated OGD-responsive endothelial lncRNAs (202). Studies have shown that MALAT1 promotes the proliferation of BMEC through various pathways such as binding to miR-145, participating in the 15-LOX1/STAT3 signaling pathway, participating in angiogenesis under OGD (24). In addition, MALAT1 acts as an endogenous competitor RNA (CeRNA) of miR-26b and inhibits the expression of miR-26b, thereby upregulating ULK2 expression and promoting BMEC autophagy and survival under OGD/R conditions (203). Zhang et al. (2021a) found that MALAT1 targets miR-375, and miR-375 targets PDE4D. Overexpression of miR-375 attenuated OGD/R-induced damage in PC-12 cells by targeting PDE4D. Both low expression of miR-375 and high expression of PDE4D reversed the promoting effect of MALAT1 knockdown on SOD level and the inhibitory effect on ROS level, inflammatory factor level and apoptosis (204). Wang et al. (2020a) found that MALAT1 was upregulated in OGD/R-treated MA-C cells (astrocytes), AQP4 was a downstream target of miR-145, and MALAT1 inhibition could reduce AQP4 by stimulating miR-145 expression (25). AQP4 silencing attenuates astrocyte injury under ischemia-reperfusion conditions *in vitro*. Therefore, MALAT1 affects AQP4 expression by competitively binding to miR-145, thereby promoting cerebral ischemia-reperfusion injury, suggesting that MALAT1 may become a new therapeutic target for the treatment of cerebral ischemic stroke. In conclusion, MALAT1 can inhibit endothelial cell apoptosis and inflammation, promote endothelial cell proliferation, angiogenesis and autophagy survival, thereby reducing the damage of hypoxia and hypoglycemia (25). It is predicted that MALAT1 has protective and healing properties in ischemic stroke injury, and may become a biomarker for cerebrovascular disease treatment and prognosis.

### 4.3 Mechanism of long non-coding RNA ANRIL in the cell cycle kinase inhibitor 4 locus involved in stroke neuroinflammation

The coding gene of ANRIL is located on chromosome 9p21, which is an antisense non-coding RNA with a size of about 3.8kb. ANRIL not only regulates cell proliferation and apoptosis in myocardial models with insufficient blood oxygen supply (205), but also is closely related to the pathological process of ischemic brain tissue. In a rat model of cerebral infarction, significantly elevated ANRIL (1.5 times more than the normal control group) activates the IκB/NF-κB pathway and upregulates vascular endothelial growth factor (VEGF) to promote angiogenesis (206). Deng (2021) found that acute cerebral ischemia-reperfusion injury can lead to increased ANRIL expression, and down-regulation of ANRIL can reduce acute ischemia-reperfusion-induced injury and neuroinflammation by inhibiting the expression of NF-κB (207). Down-regulation of ANRIL may attenuate acute cerebral ischemia-reperfusion-induced injury and neuroinflammation by regulating the miR-671-5p/NF-κB signaling pathway, suggesting that ANRIL may be a potential target for the treatment of ischemic stroke. Feng et al. (2019) showed that the expression of ANRIL in the plasma of patients with acute ischemic stroke was decreased, and the decreased levels of high-sensitivity C-reactive protein, tumor necrosis factor-α and IL-6 were negatively correlated with ANRIL levels, while elevated IL-10 levels were positively correlated with it, suggesting that ANRIL may play an anti-inflammatory role by regulating the expression of inflammatory cytokines (27). In the Han population in China mainland, Yang et al. (2018a) showed that the expression of ANRIL in patients with ischemic stroke was higher than that in the control group, and ANRIL variants rs2383207 and rs1333049 were significantly associated with the risk of ischemic stroke in men. These results suggest that ANRIL may be involved in the process of cerebral ischemia and hypoxia injury, inhibit inflammatory response, and promote angiogenesis, and the genetic polymorphism of ANRIL is also associated with ischemic stroke (208).

### 4.4 Mechanism of H19 involved in stroke neuroinflammation

H19 is an imprinted gene with a size of 2.3 kb and is only expressed in the maternal allele. The expression level of H19 was significantly increased in MCAO, OGD and OGD/R models, and it was involved in the pathophysiological process of ischemic stroke through processes involving neuronal apoptosis, neuroinflammation, and neurogenesis (18, 209). Currently, a small number of studies have begun to address the association of H19 genetic variants with ischemic stroke risk. A study showed that compared with the CC+CT genotype of H19 rs217727, the TT genotype was associated with a 1.519-fold increased risk of ischemic stroke (210). This effect was more pronounced in ischemic small vessel stroke, with a 1.941-fold increase (210). Wang et al. (2017) found that H19 knockdown blocked oxygen-glucose deprivation-driven M1 microglial polarization, decreased tumor necrosis factor-α and CD11b production, and increased Arg-1 and CD206 expression. Furthermore, H19 knockdown reversed the oxygen-glucose deprivation-induced upregulation of HDAC1 and downregulation of acetyl-histone H3 and acetyl-histone H4 (28). In contrast, HDAC1 overexpression negated the effect of H19 knockdown. Further studies showed that lncRNA H19 levels were increased in plasma and brain tissue in middle cerebral artery occluded mice (28). Lateral ventricle injection of small interfering RNA H19 reduced infarct volume and cerebral edema, decreased pro-inflammatory cytokine levels, and increased plasma anti-inflammatory cytokine interleukin-10 levels 24 h after stroke (28). This suggests that H19 is involved in the inflammatory attack in the acute phase of ischemic stroke. Furthermore, inhibition of H19 reduces HDAC1 to promote microglial polarization from M1 to M2 (the mechanism is that H19 promotes neuroinflammation by driving histone deacetylase I-dependent microglial polarization). Inhibition of H19 promotes brain IL-10 production and reduces brain TNF-α and IL-1β levels in ischemic mice. Therefore, reducing H19 can suppress neuroinflammation in ischemic stroke (28).

### 4.5 Mechanism of NKILA involved in stroke neuroinflammation

NKILA is a newly discovered lncRNA. It interacts with nuclear factor-kappa gene binding/inhibitory kappa gene binding (NF-κB/IκB) to form a stable complex, blocking the activation of transcription factor NF-κB. The NF-κB signaling pathway is a key pathway involved in the regulation of physiological and pathological mechanisms such as inflammation, immunity, and cell survival (211). Studies have shown that NKILA upregulation in OGD/R-treated neuronal cells and lentiviral vector overexpression of NKILA both inhibit NF-κB signaling and increase neuronal cell death. Conversely, shRNA silencing of NKILA almost reversed OGDR-induced NF-κB inhibition, thereby significantly attenuating neuronal cell viability reduction, apoptosis, and necrosis. The mechanism may be that the down-regulation of miR-103 and miR-107 under OGD/R conditions induces the up-regulation of NKILA in neuronal cells, thereby inhibiting NF-κB signaling and mediating neuronal cell death (29). *In vivo* experiments, Gao et al. (2021) found that NKILA significantly reduced cerebral infarct volume, brain water content and neurological function scores induced by MCAO/R. Furthermore, NKILA blocked the activation of the NF-κB pathway and inhibited astrocyte proliferation and neuronal apoptosis as well as inflammation and oxidative stress. *In vitro*, NKILA significantly inhibited the NF-κB pathway in HT22 cells. In addition, NKILA can attenuate the inflammatory response and oxidative stress of U251 cells mediated by HT22 cells after OGD/R, promote the proliferation of U251 cells and inhibit their apoptosis (212). It can be seen from the above that the NKILA/NF-κB pathway in ischemic stroke has a significant impact on the viability of neurons, and is expected to become a sensitive indicator for measuring ischemic nerve cell damage and astrocyte inflammatory oxidative stress. It is of great significance to further explore the potential mechanism of this pathway and seek specific targets for the diagnosis and treatment of ischemic nerve cell injury.

### 4.6 Mechanism of small nucleolar RNA host gene 14 involved in stroke neuroinflammation

SNHG14 is located within the Prader-Willi critical region and generates a long, spliced paternally imprinted RNA that starts from a common upstream promoter region shared by the SNRPN (small nuclear ribonucleoprotein N) and SNURF genes (213). This transcript serves as the host RNA for small nucleolar RNA, C/D boxes 115 and 116 clusters. It extends in an antisense fashion to the region of the ubiquitin protein ligase E3A gene (UBE3A) and is thought to regulate the imprinted expression of UBE3A in the brain (214). Under OGD/R conditions, propofol PPF inhibited the expression of SNHG14 through the p38 MAPK signaling pathway. SNHG14 promotes the expression of Atg5 and Beclin 1 by sponging miR-30b-5p, thereby activating autophagy and aggravating CI/R injury (30). In lipopolysaccharide (LPS)-induced PC-12 cells, NHG14 knockdown alleviated LPS-induced PC-12 cell inflammation and apoptosis by regulating miR-181b-5p (215). Deng et al. (2020a) found that the lncRNA SNHG14 induced excessive mitophagy through the miR-182-5p/BINP3 axis in HT22 mouse hippocampal neuronal cells, thereby promoting OGD/R-induced neuronal damage. Therefore, the SNHG14/miR-182-5p/BINP3 axis may be a valuable target for CI/R injury therapy (216). SNHG14 is highly expressed in ischemic brain tissue and primary microglia after oxygen and glucose deprivation treatment. High expression of SNHG14 increases the expression of lipoprotein-related phospholipase by inhibiting miR-145-5p, which promotes the release of a large number of inflammatory cytokines (TNF-α, NO) after ischemia and aggravates neuronal damage (31). Li (2022) found that LncRNA SNHG14 was highly expressed in stroke patients. LncRNA SNHG14 induces inflammation in an *in vitro* stroke model by inhibiting the miR-124-3p/TRAF6 axis. In addition, down-regulation of LncRNA SNHG14 attenuated inflammation in an *in vitro* stroke model by inducing the miR-124-3p/TRAF6 axis (217). Bu et al. (2021) found that silencing SNHG14 could significantly promote OGD-induced primary cortical neuron proliferation, inhibit apoptosis and inflammatory responses, and alleviate neuronal damage. The mechanism is that SNHG14 promotes neuronal damage by regulating the miR-181c-5p/BMF axis, suggesting that SNHG14 may be a potential target to alleviate IS-induced brain damage (218). Zhang et al. (2021b) found that SNHG14 was up-regulated in the MCAO mouse model. Deletion of SNHG14 attenuates cerebral ischemia in MCAO model mice (32). SNHG14 silencing suppresses inflammation and oxidative stress in OGD-exposed BV2 cells. After OGD treatment of BV2 cells, the level of miR-199b was decreased, while the level of AQP4 was increased. Knockdown of miR-199b reversed the effect of SNHG14 knockdown on OGD-stimulated ischemic injury in BV2 cells. Furthermore, AQP4 overexpression abolished the effect of miR-199b on ischemic injury in OGD-treated BV2 cells. Furthermore, SNHG14 indirectly regulated the expression of AQP4 by inhibiting miR-199b. Knockdown of SNHG14 suppresses inflammation and oxidative stress *via* the miR-199b/AQP4 axis and alleviates ischemic brain injury (32). Zhong et al. (2019) found that SNHG14 was up-regulated in OGD/R-treated PC-12 cells in MCAO/R-treated brain tissue. Interference of SNHG14 with shRNA vectors enhanced neuronal survival and suppressed inflammation in response to OGD/R injury. SNHG14 positively regulates the expression of Rho-associated coiled-coil-containing protein kinase 1 (ROCK1) by acting as a sponge for microRNA (miR)-136-5p. SNHG14 promotes neural injury and inflammatory responses by increasing ROCK1 expression while reducing miR-136-5p levels in OGD/R-induced injury (33).

### 4.7 Mechanism of SNHG15 involved in monocyte-macrophage inflammation in stroke

At present, more and more studies have shown that lncRNAs are involved in the central and peripheral immune systems after stroke (219, 220). Stroke can lead to suppression of the peripheral immune system, which can lead to the development of post-stroke infections (221). Sun et al. (2022) found that SNHG15 was significantly highly expressed in monocytes in PBMCs of stroke patients, and IL-4 promoted the expression of SNHG15, LPS inhibited the expression of SNHG15, and SNHG15 could partially inhibit the transformation of macrophages to M1 type. This suggests that SNHG15 is closely related to the polarization of macrophages. Therefore, IncRNASNHG15 promotes the differentiation of macrophages into M2 type, which in turn inhibits the peripheral immune response, induces post-stroke immunosuppression, and leads to the occurrence of stroke-related infection (34). The mechanism is that SNHG15 participates in regulating the polarization of macrophages by promoting the synthesis of p-STAT6 in monocytes and macrophages, and the p-STAT6 after entering the nucleus binds to the promoter region of SNHG15. This further promotes the expression of SNHG15, thereby promoting the differentiation of macrophages to M2 type, which in turn aggravates post-stroke immunosuppression and leads to stroke-related infection. Guo et al. (2020) established an *in vivo* mouse MCAO model and an *in vitro* neurogenic mouse cell line Neuro-2a (N2a) for an OGD model. They identified SNHG15 silencing through sequestration of miR-18a and subsequent activation of ERK/MEK leading to upregulation of CXCL13, thereby enhancing viability while reducing apoptosis in N2a cells (222). By establishing an *in vitro* cerebral ischemia-reperfusion injury model of OGD/R-induced neuronal injury, SNHG15 silencing blocked the effects of OGD/R treatment on cell viability, apoptosis, inflammation, and oxidation in PC12 cells. However, these effects were restored after transfection with miR-455-3p inhibitor. Furthermore, SNHG15 acts as a sponge for miR-455-3p and miR-455-3p bound to TP53INP1. SNHG15 promotes OGD/R-induced neuronal damage by regulating the miR-455-3p/TP53INP1 axis, which provides new insights into the study of lncRNA-guided ischemic stroke therapy (223). Hu et al. (2021) found that SNHG15 was up-regulated in hypoxia/ischemia mouse or cell models by inducing hypoxia/ischemia model by mouse MCAO and OGD/R. Inhibition of SNHG15 ameliorated ischemia/hypoxia-induced neuronal damage and microglial inflammation by regulating the miR-302a-3p/STAT1/NF-κB pathway (35).

### 4.8 Mechanism of LncRNA SNHG4 involved in stroke microglial inflammation

In ACI, hyperactivated microglia produce large amounts of inflammatory cytokines, causing an inflammatory storm and ultimately exacerbating disease progression (224). Zhang et al. (2020a) constructed an *in vivo* model of middle cerebral artery occlusion (MCAO) in rats and used LPS-induced and oxygen-glucose deprivation methods to simulate the activation of microglia *in vitro*. Compared with the control group, the expression of SNHG4 derived from microglia in the ACI patient samples and the MCAO group was significantly down-regulated, while the expression of miR-449c-5p was significantly up-regulated. Overexpression of SNHG4 and knockdown of miR-449c-5p inhibited the expression of pro-inflammatory cytokines and promoted the expression of anti-inflammatory cytokines in microglia. Meanwhile, phosphorylated-STAT6 was upregulated, whereas knockdown of SNHG4 and overexpression of miR-449c-5p in microglia had opposite effects. Therefore, SNHG4 regulates STAT6 and inhibits inflammation by adsorbing miR-449c-5p in microglia during cerebral ischemia-reperfusion injury (36).

### 4.9 Mechanism of LncRNA Nespas involved in stroke microglial inflammation

Nespas is one of the factors of LncRNA. Deng et al. established a mouse MCAO model, and their experiments confirmed that LncRNANespas played an important protective role in ischemic stroke by reducing the occurrence of neuroinflammation (37). Among them, LncRNA Nespas was abundantly expressed in ischemic brain tissue of MCAO mice and BV2 cells undergoing OGD. High expression of Nespas impedes the activation of transforming growth factor-beta-activated kinase 1 (TAK1) by eliminating the interaction between tripartite motif 8 (TRIM8) and TAK1. Overexpressed Nespas restricts NF-κB activation *via* TAK1, which inhibits microglial cell death and neuroinflammation in the ischemic microenvironment. Furthermore, Nespas silencing in MCAO mice worsens ischemic brain injury (37).

### 4.10 Mechanism of LncRNA ZFASI involved in stroke inflammation

LncZFAS1 is located on chromosome 20q13.13 and is an IncRNA transcribed from the antisense strand near the 5’ end of the protein-coding gene Znfx1. It has three C/D box snoRNAs (SNO RDs): Snord12, Snord12b and Snord12c (225, 226). LncZFAS1 was originally identified as a regulator of mammary alveolar development and epithelial cell differentiation (227). This study found that IncZFAS1 was highly expressed in esophageal cancer tissues and cells. Plasma lncRNA ZFASI levels are downregulated in patients with ischemic cerebral infarction. Overexpression of ZFAS1 alleviated neurological deficits and neuronal damage in MCAO/R model rats; and overexpression of ZFAS1 reduced the expression of pro-apoptotic factors Bax and caspase-3 in OGD/R PC12 cells, and increased the expression of anti-apoptotic factor Bcl2. It can also reduce the release of pro-inflammatory factors TNF-α, IL-1β and monocyte chemoattractant protein-1 (MCP-1) by negatively regulating miR-582-3p (38).

### 4.11 Mechanism of LncRNA MIAT involved in stroke inflammation

MIAT, also known as RNCR2 or Gomaful8,91, is one of the long non-coding RNAs associated with myocardial infarction discovered early, which is located on human chromosome 22q12.1 with a length of 30051 bp (228). Ishii et al. (2006) conducted a large-scale case-control study using the single nucleotide polymorphisms (SNP) marker of the 52608 haplotype and found that a complete new gene could be isolated in the susceptible site of myocardial infarction, which was named MIAT. The results of *in vitro* protein translation analysis showed that MIAT did not encode any protein, just a functional RNA (229). Furthermore, MIAT belongs to the nuclear-retained Lnc RNAs. It is located in a new subnuclear domain and is punctately distributed throughout the nucleus, is associated with the nuclear matrix, contains 7 exons, and can also be spliced to produce more than a dozen isoforms. MIAT is highly conserved in placental mammals, and it is not only highly expressed in fetal and mouse brains, but also persistently expressed in adult brain neurons (230). Zhu et al. (2018) showed that MIAT may be a potential diagnostic and prognostic indicator of IS. Deng et al. (2020b) showed that inhibition of MIAT reduced CMEC injury, induced CMEC angiogenesis, increased the number of surviving neurons, promoted miR-204-5p expression and suppressed HMGB1 expression in OGD-treated CMECs. MIAT promotes HMGB1 expression by competitively binding to miR-204-5p, thereby regulating the damage of CMECs after cerebral ischemia (231). Zhang et al. (2021c) showed that the expression level of MIAT in serum of patients with ischemic stroke was higher than that of normal people, and down-regulation of MIAT could reduce neurological damage and brain tissue damage in MCAO/R mice. MIAT can act as an endogenous competing RNA to negatively regulate microRNA-874-3p to reduce the level of pro-inflammatory factor IL-1β and reduce the neuroinflammatory response induced by cerebral ischemia/reperfusion (39).

### 4.12 Mechanism of IncRNA taurine up-regulated gene 1 involved in stroke inflammation

LncRNA TUG1 is highly conserved, located on chromosome 22q12.2, and its gene sequence consists of 7598 nucleotides (232). A spliced, polyadenylated-tailed lncRNA was first discovered in neonatal mouse retinal cells cultured *in vitro*. Since the expression of IncRNA TUG1 is up-regulated with the addition of taurine, it is called “taurine up-regulated gene 1” (233). Taurine is a sulfur-containing amino acid that is mainly produced in the liver and kidneys, and is present in organs such as the retina, brain, heart, and placenta. It plays a key role in brain development, optical and immune systems, and biological processes such as osmoregulation, reproduction, membrane stabilization, myocardial regulation, and inflammation (234, 235). Current research shows that TUG1 is widely expressed in the adult brain. Chen et al. (2017b) reported that LncRNA TUG1 can promote neuronal apoptosis under ischemic conditions. It is speculated that LncRNA TUG1 may play a role in the neurological injury caused by ischemic stroke (236). Zhang et al. (2021c) found that LncRNA TUG1 regulates mitogen-activated protein kinase-activated protein kinase 2 (MK2) by inhibiting the expression of miR-137. MK2-mediated inflammatory response, thereby promoting nerve damage in rats with focal cerebral ischemia (237). Wang et al. (2019) found that the level of TUGI was up-regulated in microglia after OGD/R treatment. UG1 knockdown promoted the transformation of microglia from M1-like phenotype to M2-like phenotype, and reduced the production of pro-inflammatory cytokines (TNF-α, IL-6) and anti-inflammatory cytokines (IL-10), thus promoting the survival of SH-SY5 Y cells. Meanwhile, TUG1 knockdown also prevented OGD-induced activation of the NF-κB pathway, as manifested by decreased ratios of p-p65/p65 and p-IκBα/IκBα proteins. Furthermore, we found that TUG1 could physically associate with miR-145a-5p, and miR-145a-5p inhibitor abrogated the protective effect of TUG1 knockdown by activating the NF-κB pathway. This suggests a negative interaction between TUG1 and miR-145a-5p (40).

### 4.13 Mechanism of IncRNA MEG3 involved in stroke inflammation

MEG3 is about 1.6 kb long and is located on human chromosome 14q32.3 (238). In a cerebral ischemia-reperfusion model, MEG3 expression is elevated and is considered to be an injury factor. Down-regulation of MEG3 expression with the small interfering RANA plasmid si-MEG3 reduced cerebral infarct volume, reduced edema, and decreased neurobehavioral scores. Up-regulation of MEG3 increases neuronal loss and apoptosis, which is thought to be directly combined with P53 to promote neuronal apoptosis. The separation of MEG3 and P53 reduces neuronal damage. MEG3 is also thought to induce oxidative stress by activating 2/15 lipoxygenase (12/15-LOX), leading to neuronal damage (239). Down-regulation of MEG3 can reduce cerebral ischemia-reperfusion-induced injury by regulating miR-21/PDCD4 and improve neurobehavioral function in MCAO/R mice (240). Down-regulation of MEG3 can reduce cerebral ischemia-reperfusion pyroptosis and neuroinflammation through the miR-485/AIM2 signaling pathway, down-regulate the expression of AIM2 in the inflammasome. It activates caspase1 signaling during brain I/R to promote pyroptosis by targeting the MEG3/miR-485/AIM2 axis, suggesting that this axis may be an effective therapeutic target for ischemic stroke (41). Down-regulation of MEG3 can also protect human microvascular endothelial cells by activating the Notch signaling pathway, promote angiogenesis, and reduce the release of pro-inflammatory factors in neurovascular units (241). Current studies have shown that up-regulation of Krüppel-like factor 4 (KLF4) can reduce the levels of M1 markers (CD86 and iNOS), TNF-α and IL-1β, and increase the expressions of M2 markers (CD206 and Arg1), IL-10 and IL-4 in BV2 cells induced by OGD/R. Li et al. (2020a) found that MEG3 was elevated in the brain tissue of MCAO/R mice and in OGD/R-induced BV2 cells. MEG3 binds to KLF4 and inhibits its protein progression. MEG3 deletion enhanced M2 polarization and reduced M1 polarization in microglia by targeting KLF4. Ultimately, MEG3 knockdown reduces neuroinflammation to alleviate cerebral ischemia-reperfusion. Furthermore, MEG3 was overexpressed in a TBI cell model (LPS+ATP-induced microglia obtained from normal mice) (42). MEG3 targets miR-7a-5p and reduces its expression, and MEG3 also acts as a ceRNA for miR-7a-5p and inhibits the repression of NLRP3 by miR-7a-5p. Functionally, MEG3 upregulation enhances microglial activation and inflammation through the miR-7a-5p-NLRP3 axis in an *in vitro* model of TBI (242). The above suggests that MEG3 may serve as a therapeutic target for reducing the inflammatory response in patients with ischemic stroke.

### 4.14 Mechanism of LncRNA 1810034E14Rik involved in stroke microglia

LncRNA-1810034E14Rik, located on chromosome 13, was significantly reduced in microglia both after OGD induction and after MCAO. To date, there are no studies on the function of IncRNA-1810034E14Rik in any disease. Bioinformatics suggested that it may be related to innate immune response and apoptosis. LncRNA 1810034E14Rik was significantly decreased in microglia after OGD exposure. Elevated 1810034E14Rik expression impedes microglial activation and microglial inflammation *in vitro* and *in vivo*. Furthermore, upregulated 1810034E14Rik abrogated neuronal damage caused by OGD-treated microglia. Furthermore, enhanced 1810034E14Rik expression promoted motor recovery and reduced infarct volume in MCAO mice (43). OGD/R microglia LncRNA 1810034E14Rik was significantly decreased, and up-regulation of LncRNA 1810034E14Rik may reduce microglial activation and inflammatory response by inhibiting the NF-KB pathway (43).

### 4.15 Mechanism of LncRNA Maclpil (Gm15628) in stroke macrophage polarization inflammation

Wang et al. identified 73 lncRNAs that were differentially expressed in monocyte-derived macrophages (MoDM) and microglia-derived macrophages (MiDM) isolated from ischemic brain three days after stroke. Among them, the lncRNA GM15628 is highly expressed in proinflammatory MoDM but not MiDM, and is functionally related to its adjacent gene lymphocyte cytoplasmic protein 1 (LCP1). LCP1 plays a role in maintaining cell morphology and cell migration. Using cultured macrophages polarized by LPS, M(LPS), it was found that downregulation of Maclpil in M(LPS) decreased the expression of pro-inflammatory genes while promoting the expression of anti-inflammatory genes. Maclpil inhibition also reduced the migratory and phagocytic abilities of MoDMs by inhibiting LCP1. Furthermore, adoptive transfer of Maclpil silenced M(LPS), reduced ischemic cerebral infarction, improved behavioral performance and attenuated MoDM infiltration in the ischemic hemisphere. In addition, the expression level of LncRNA Maclpil (Gm15628) in the cerebral infarction area of MCAO/R mice was increased, and down-regulation of LncRNA Maclpil could reduce the inflammatory response in *in vivo* and *in vitro* models of cerebral ischemia-reperfusion. Down-regulation of Maclpil can inhibit the migration and phagocytosis of monocyte-macrophages by regulating LCP1, reduce the size of cerebral infarction in MCAO/R mice, improve neurobehavioral function, and reduce the infiltration of monocyte-macrophages in the cerebral infarct area (44).

### 4.16 Mechanism of LncRNA small nucleolar RNA host gene 8 involved in stroke inflammation

In the brain tissue of MCAO mice, the expression of SNHG8 is down-regulated, and MiR-425-5p can share a binding site with SNHG8 and sirtuin1. SNHG8 functions as a ceRNA of miR-425-5p and inhibits the effect of miR-425-5p on sirtuin1. SNHG8 overexpression attenuated miR-425-5p to increase sirtuin1 expression and inhibit NF-κB phosphorylation. SNHG8 amplification inhibits microglial activation and inflammation through the miR-425-5p/sirtuin1/NF-κB pathway, thereby reducing BMEC damage. Furthermore, overexpressed SNHG8 suppressed neuronal damage, brain edema, and neurological loss in mice after ischemic stroke. Therefore, SNHG8, as an endogenous competitive RNA, adsorbs miR-425-5p to regulate SIRTI/NF-KB to reduce the inflammatory response of cerebral microvascular endothelial cells and microglia induced by cerebral ischemia/reperfusion, and protect the blood brain barrier of MCAO/R mice (45). Liu (2020) used HT22 cells and C57BL/6 J mice to construct *in vitro* and *in vivo* models of CCI, and found that Snhg8 and Hoxa13 were down-expressed and miR-384 was over-expressed in CCI-induced mouse hippocampal neurons. Overexpression of Snhg8, overexpression of Hoxa13, and silencing of miR-384 could inhibit CCI-induced apoptosis of mouse hippocampal neurons (243). Snhg8 binds to miR-384 and negatively regulates the expression of miR-384; miR-384 targets and binds to the 3’UTR of Hoxa13 mRNA and negatively regulates the expression of Hoxa13. Hoxa13 promotes its transcriptional regulation of CCI-induced apoptosis of mouse hippocampal neurons by binding to the FAM3A promoter region. Snhg8/miR-384/Hoxa13/FAM3A plays an important role in regulating CCI-induced neuronal apoptosis (243). Zhang et al. (2021d) established a rat middle cerebral artery permanent occlusion (p-MCAO) model and a microglia OGD model, and LncRNA SNHG8 was downregulated in MCAO rats. Overexpression of SNHG8 ameliorated neurological deficits, decreased brain water content, BBB permeability, brain tissue damage and inflammation, and inhibited microglia activation. In OGD-induced microglia, overexpression of SNHG8 or downregulation of miR-449c-5p increased cell viability and decreased lactate dehydrogenase activity. Furthermore, SNHG8 uptakes miR-449c-5p to regulate SIRT1. Overexpression of SNHG8 increased the expression of SIRT1 and FoxO1. MiR-449c-5p mimic can abrogate the effects of SNHG8 overexpression on ischemic microglia. In conclusion, SNHG8 inhibits microglial activation and BBB permeability through the miR-449c-5p/SIRT1/FoxO1 pathway, thereby triggering protection against ischemic brain injury (46).

### 4.17 Mechanism of LncRNA NEAT1 involved in stroke inflammation

NEATI is enriched in the nucleus, about 3.2kb long, and is a long non-coding RNA critical for the formation and maintenance of nuclear substructure paraspeckle (244). Current studies have found that NEATI plays an important role in the inflammatory response (245). Jin (2021) confirmed by bioinformatics analysis and experiments that Neat1 promotes the activation of microglia and promotes inflammatory response in the pathological process of ischemic stroke. Knockdown of Neat1 significantly inhibited microglial activation, decreased the release of pro-inflammatory cytokines IL-1β, IL-6 and TNF-α, increased the levels of anti-inflammatory cytokines IL-4 and IL-10, decreased the expression of apoptotic pathway proteins and decreased the number of neuronal apoptosis. In addition, knockdown of Neat1 can also inhibit the activity of the downstream JAK-STAT pathway closely related to inflammation and the up-regulated expression of HMGB1 (47). In summary, lncRNA-Neat1 is abnormally up-regulated in MCAO, which activates microglia and plays a pro-inflammatory role, and knockdown of Neat1 can improve the inflammatory response after ischemic stroke, reduce neuronal apoptosis and improve neurological function. Jin et al. (2021) showed through *in vivo* experiments that Neat1 has abnormally high expression after MCAO. Knockdown of Neat1 can significantly alleviate brain injury by reducing the number of activated microglia and reducing the release of pro-inflammatory cytokines (246). In terms of macrophages, Wang and Guo (2020) found that the lncRNA NEAT1 promoted macrophage M2 polarization through the miR-125a-5p/TRAF6/TAK1 axis, thereby improving LPS-induced inflammatory responses (247). YY1-induced upregulation of lncRNA NEAT1 promotes OGD/R injury-induced brain microglial inflammatory response through the Wnt/β-catenin signaling pathway (48). Zhang (2019) found that long non-coding RNA Neatl may be bound to the NLRP3 inflammasome by NLRP3 UV cross-linking immunoprecipitation combined with high-throughput sequencing (248). Further studies have found that Neatl in mouse macrophages can bind to classical inflammatory bodies such as NLRP3, NLRC4 and AIM2, and promote the assembly and activation of these inflammatory bodies, thereby promoting the activation of caspase-1, cytokine maturation and cell pyroptosis in the downstream. Gastrodin attenuates I/R-induced neuronal inflammatory responses by regulating the lncRNA NEAT1/miR-22-3p axis, and significantly attenuates brain I/R injury (49). Lian and Luo (2021) established the OGD/R injury of CHME5 cells as an *in vitro* stroke model. They found that knockdown of NEAT1 modulates OGD/R injury in CHME5 cells *via* the miR-374a-5p/NFAT5 axis, thereby inducing microglia migration from M1 to M2 and suppressing inflammatory responses, making it a potential target for stroke therapy (249). Ni et al. (2020) induced OGD/R *in vitro* to mimic cerebral ischemia-reperfusion injury and found that the lncRNA NEAT1 may inhibit the polarization of microglia toward the M1 phenotype to reduce OGD/R-induced damage and reduce the activity of the AKT/STAT3 pathway (50). In summary, the lncRNA NEAT1 may be a potential target for new therapeutic interventions in CIRI.

### 4.18 Mechanism of lncRNA OIP5-AS1 involved in stroke inflammation

OIP5-AS1, a long non-coding RNA on human chromosome 15q15.1 with a length of 8844 bp, is transcribed in the opposite direction to OIP5 (250). Interestingly, a recent study found that microangiopathy in diabetic mice was significantly aggravated with downregulation of OIP5-AS1, accompanied by neurological deficits (251). Chen et al. (2021b) modeled *in vitro* ischemic penumbra and OGD/R-treated microglia in MCAO/R-injured rats. They observed a significant increase in infarct volume, neuronal apoptosis, inflammation, and oxidative stress responses in infarcts of MCAO/R rats, consistent with down-regulation of OIP5-AS1 and CTRP3 levels and up-regulation of miR-186-5p (252). Functional studies showed that upregulation of OIP5-AS1 attenuated infarct volume, neuronal apoptosis, microglia/macrophage inflammation and oxidative stress responses induced by MCAO/R or OGD/R. Mechanistically, they found that the OIP5-AS1-miR-186-5p-CTRP3 axis plays an important role in regulating microglia/macrophage activation and neuronal apoptosis. Zheng et al. (2021) found that the expression of OIP5-AS1 was up-regulated in ox-LDL-induced human umbilical vein endothelial cells (HUVECs) model, while miR-98-5p was down-regulated in ox-LDL-induced human umbilical vein endothelial cells (HUVECs) (51). Functionally, knockdown of OIP5-AS1 induced ox-LDL-induced HUVEC cell proliferation and inhibited apoptosis, inflammatory damage, and oxidative stress damage. Interestingly, miR-98-5p was a target of OIP5-AS1, and miR-98-5p inhibition abolished the effect of OIP5-AS1 downregulation on ox-LDL-induced damage in HUVECs. More importantly, miR-98-5p directly targets HMGB1, and OIP5-AS1 regulates HMGB1 expression by sponging miR-98-5p. OIP5-AS1 can regulate the TLR4/nuclear factor-κB (NF-κB) signaling pathway through the miR-98-5p/HMGB1 axis. LncRNA OIP5-AS1 accelerates ox-LDL-induced endothelial cell injury by regulating miR-98-5p-mediated HMGB1 through TLR4/NF-κB signaling pathway.

### 4.19 Mechanism of LncRNA FIRRE involved in stroke

Zang et al. (2018) constructed an OGD/R injury model of brain microglia, performed microarray analysis and analyzed the association of lncRNA functional intergenic repeat RNA elements (FIRRE) with OGD/R injury. Based on molecular biotechnology, we demonstrated that FIRRE can activate the NF-kB signaling pathway. Meanwhile, activated NF-kB promoted the expression of FIRRE in OGD/R-treated brain microglia. Thus, FIRRE and NF-kB form a positive feedback loop to promote transcription of the NLRP3 inflammasome, which leads to OGD/R injury in brain microglia. This LncRNA FIRRE may be a new and specific therapeutic target for potential ischemic stroke in the future (52).

### 4.20 Mechanism of LncRNA rhabdomyosarcoma 2-related transcript involved in stroke inflammation

Cheng et al. (2020) found that the RMST/hnRNPK/p53/miR-107/Bcl2l2 axis plays an important role in regulating neuronal apoptosis in the OGD-treated HT-22 hippocampal neuron cell line model group (53). Altered RMST expression resulted in dramatic changes in HT-22 cell proliferation and apoptosis. Mechanistically, RMST indirectly activates the p53/miR-107 signaling pathway by interacting with heterogeneous nuclear ribonucleoprotein K (hnRNPK) and exerts its pro-apoptotic function in HT-22 cells. Yin et al. (2021) established an *in vitro* ischemic stroke model by treating cerebral microvascular endothelial cells with OGD. MST knockdown was found to attenuate OGD-induced HBMEC and bEnd.3 cell damage by modulating the miR-204-5p/VCAM1 axis, suggesting a possible therapeutic strategy for future ischemic stroke therapy (253). Sun et al. (2019) established an OGD cerebral ischemic stroke cell model and found that in OGD-stimulated BV2 microglia, RMST was highly expressed, M1 expression increased, and M2 marker expression decreased. Functional experiments found that RMST promotes OGD-induced M1 polarization in BV2 cells. RMST activates the TAK1-NF-κB signaling pathway through competitive binding with hnRNPK in BV2 cells. RMST promotes OGD-induced microglial M1 polarization through the hnRNPK-TAK1-NF-κB axis. RMST increases OGD-induced neuronal apoptosis by modulating microglial polarization, which will provide a basis for understanding the pathogenesis of cerebrovascular disease (54).

### 4.21 Mechanism of LncRNA XIST involved in stroke inflammation

Wang et al. (2021a) found that XIST was elevated in the ischemic penumbra of OGD/R model-treated PC12 cells as well as mice with MCAO/R (55). Knockdown of XIST promoted cell survival, inhibited apoptosis, and attenuated inflammatory damage in OGDR PC12 cells *in vitro*. *In vivo*, inhibition of XIST significantly reduced nerve damage, promoted neuronal proliferation, and inhibited apoptosis in MCAO mice. Mechanistically, XIST acts as a competing endogenous RNA for miR-362 to regulate the downstream gene ROCK2. In conclusion, depletion of XIST attenuated I/R-induced neural damage and inflammatory responses through the miR-362/ROCK2 axis. Zhang et al. (2021f) found that XIST expression was upregulated in the brain tissue of the I/R mouse model and in OGD/R-treated neural 2a (N2a) cells. Knockdown of XIST attenuated brain damage and reduced N2a cell apoptosis and reactive oxygen species (ROS) production. In addition, luciferase reporter and RNA immunoprecipitation assays determined that XIST can bind to the microRNA miR-27a-3p (254). They found that the expression of miR-27a-3p was down-regulated in the brain tissue of the I/R mouse model and in OGD/R-induced N2a cells. Furthermore, miR-27a-3p overexpression attenuated I/R-induced brain injury and inhibited apoptosis and ROS production in N2a cells, finding that miR-27a-3p targets FOXO3. Silencing of FOXO3 attenuated brain injury and inhibited N2a cell apoptosis and ROS production. These findings suggest that XIST aggravates brain I/R injury by regulating miR-27a-3p/FOXO3 signaling (254). Guo et al. (2022) found that protocatechuic aldehyde (PCA) reduced cerebral infarct volume in MCAO rats and promoted cell survival and proliferation in OGD/reperfusion-treated rBMECs, reversing pyroptosis. Furthermore, PCA enhanced the antioxidant activity and mitochondrial membrane potential of rBMECs. PCA also enhanced the expression of lncRNA Xist, and when the expression of lncRNA Xist was silenced, PCA could not well alleviate the pyroptosis in rBMECs (255). Wang et al. (2021b) found that the expression of lncRNA XIST in patients with acute ischemic stroke, MCAO mice and OGD/R model decreased in the early stage of acute ischemic stroke, but increased later in patients with acute ischemic stroke and OGD/R model (56). Furthermore, serum levels of lncRNA XIST were negatively correlated with the severity of neurological damage in patients with acute ischemic stroke. Further studies revealed that lncRNA XIST regulates the expression of pro-angiogenic factor-integrin α5 (Itgα5) and anti-inflammatory factor-Kruppel-like transcription factor 4 (KLF4) by targeting microRNA-92a (miR-92a). Silencing of lncRNA XIST impairs angiogenesis after CIS and exacerbates cerebrovascular injury, resulting in larger infarcts and more severe neurological deficits in mice with transient MCAO. Mechanistic analysis showed that lncRNA XIST regulates angiogenesis after acute ischemic stroke and alleviates cerebrovascular injury by mediating miR-92a/Itgα5 or KLF4 axis, respectively (56).

### 4.22 Mechanism of LncRNA Gm4419 involved in stroke inflammation

Wen et al. (2017) found that the lncRNA Gm4419 acts as a key mediator of the activation of NF-κB signaling pathway, causing neuroinflammatory damage during OGD/R. Gm4419 is abnormally upregulated in OGD/R-treated microglia. Among them, high levels of Gm4419 promote the phosphorylation of IκBα by physically binding to IκBα, resulting in increased levels of nuclear NF-κB transcriptionally activated by TNF-α, IL-1β, and IL-6. In addition, knockdown of Gm4419 acts as a NF-κB inhibitor in OGD/R microglia, suggesting that downregulation of Gm4419 has a protective effect on OGD/R injury. In conclusion, Gm4419 is required for microglial OGD/R injury through activation of NF-κB signaling, suggesting that Gm4419 appears to be a promising therapeutic target for ischemic stroke (57).

**Figure 3:**
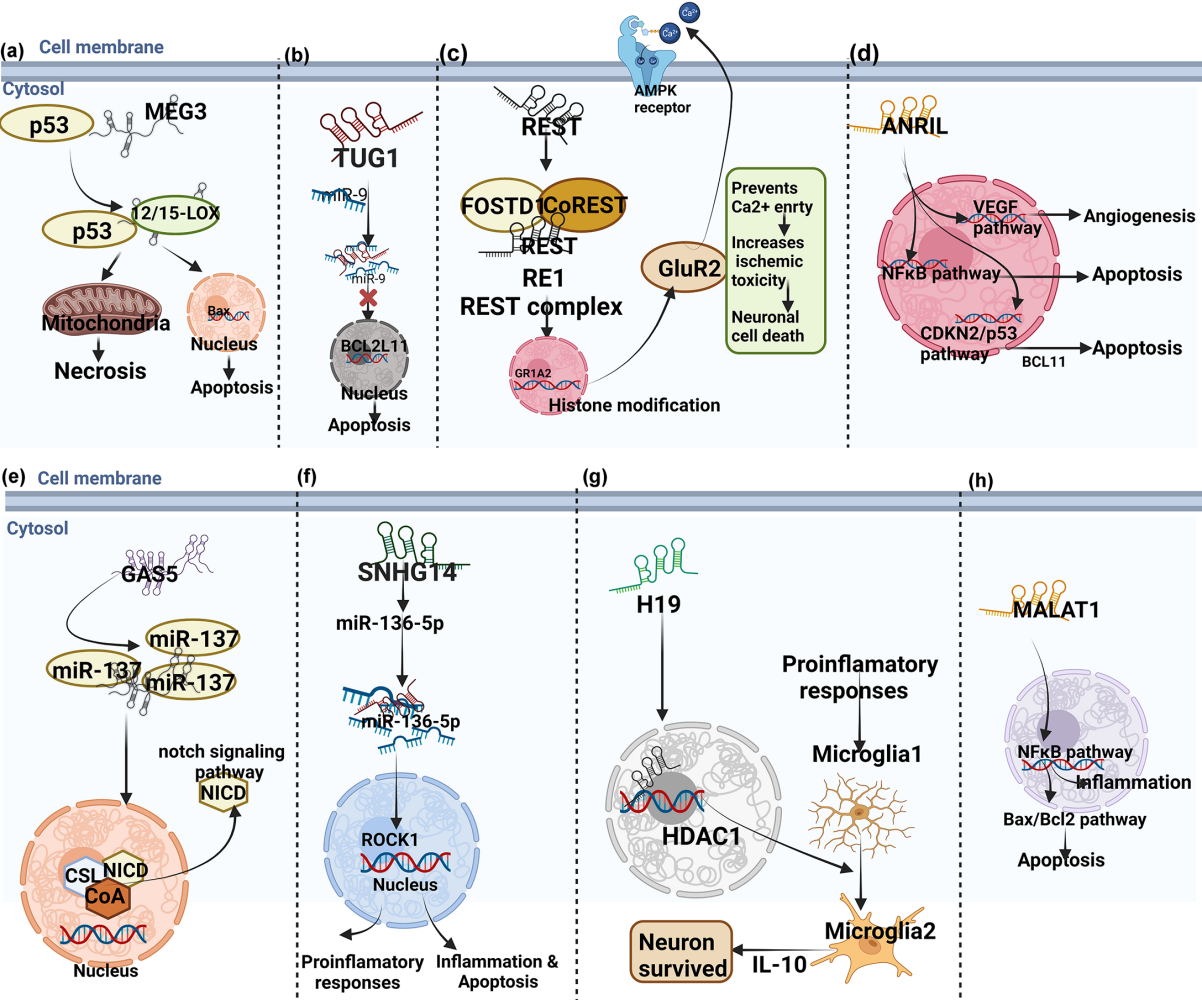
The mechanisms of lncRNAs in cerebral ischemia/ischemia-reperfusion injury. (**A–H** are schematic diagrams of the mechanism of several lncRNAs regulating neuroinflammation in regulating neuroimmune inflammation in cerebral ischemia/ischemia-reperfusion injury).

**Figure 4:**
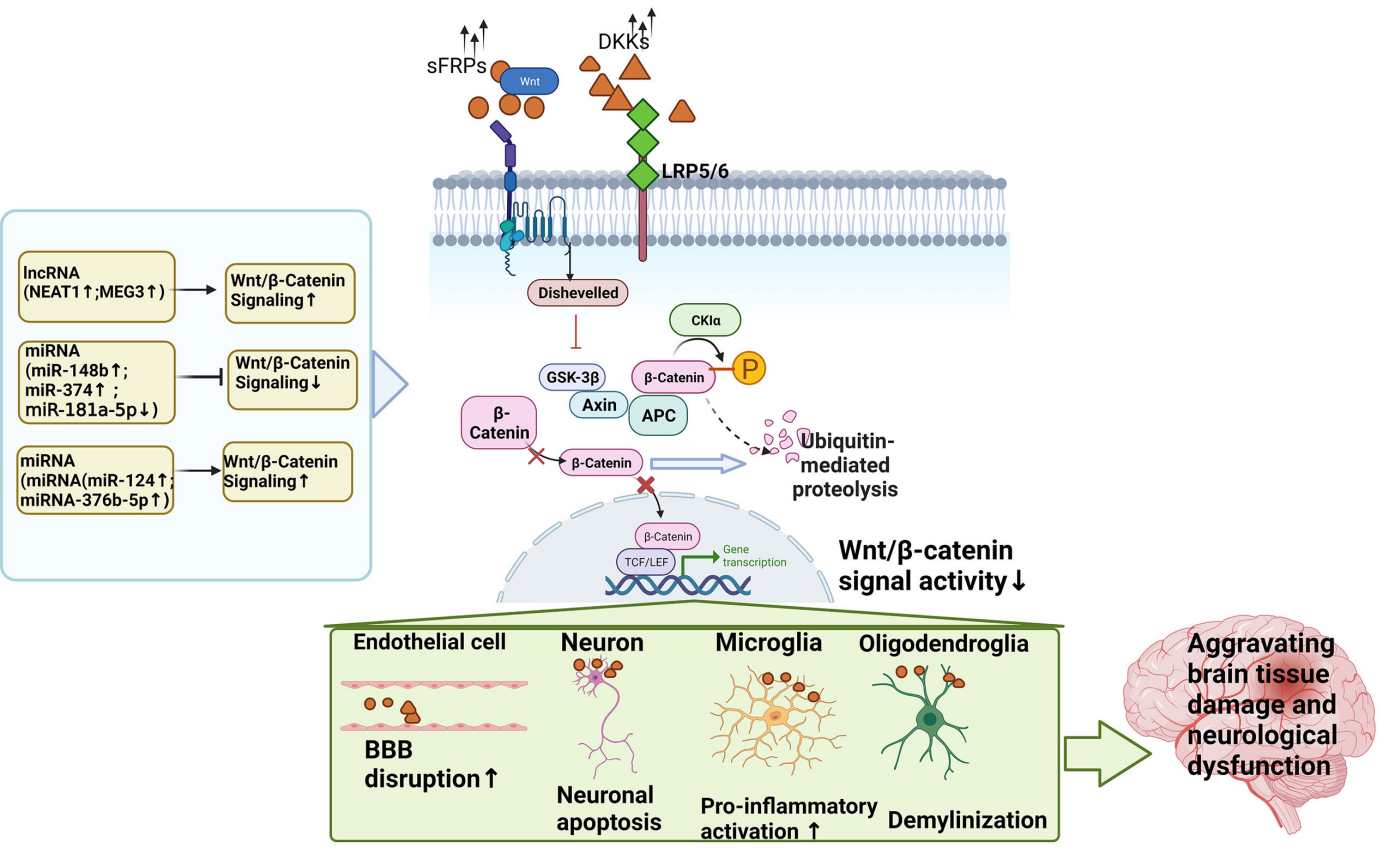
The action mode of ncRNAs in the Wnt signaling pathway.

**Figure 5:**
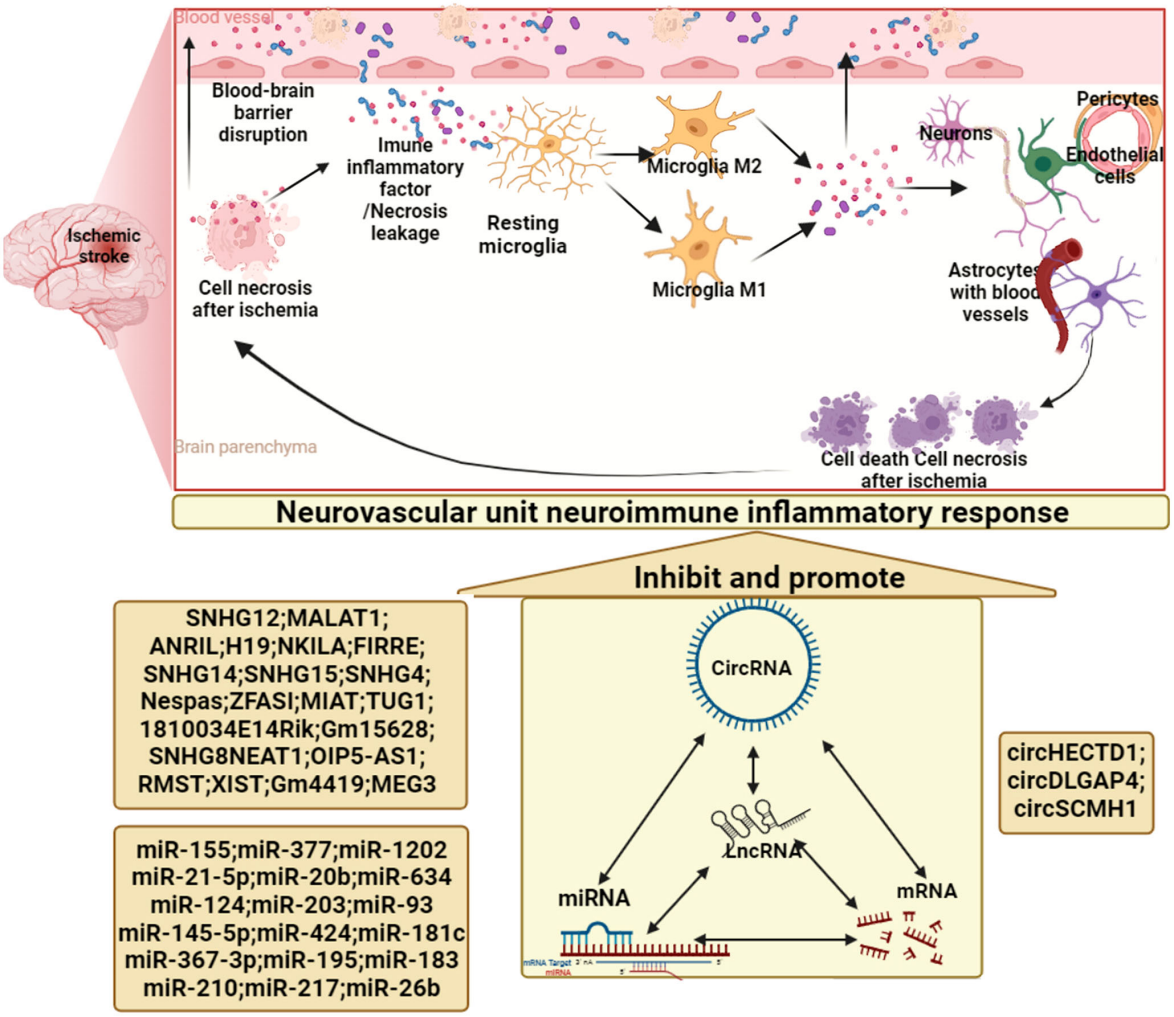
Non-coding RNA in neuroinflammation and immune regulation in cerebral infarction/ischemia-reperfusion injury.

In conclusion, LncRNA is closely related to neuroinflammation in ischemic stroke, and has the potential to become a biomarker and therapeutic target for neuroinflammation in ischemic stroke. It overcomes the clinical difficulties of early diagnosis, short treatment window and poor prognosis, and provides scientific basis for the diagnosis and treatment of ischemic stroke. However, the correlation between lncRNAs and ischemic stroke is still in the preliminary stage and has broad research prospects, and in-depth study of its mechanism is the future development trend. The mechanisms of lncRNAs in cerebral ischemia/ischemia-reperfusion injury were summarized in **Table 1** and shown in **Figures 3**, **5**. Taking the Wnt signaling pathway as an example, **Figure 4** shows the action mode of lncRNAs in the Wnt signaling pathway.

## 5 Mechanisms of miRNAs involved in neuroinflammation and immune regulation in cerebral infarction/ischemia-reperfusion injury

miRNAs are small non-coding RNAs of 19-22 nucleotides in length, which bind to the 3’ untranslated region of messenger RNA and mediate post-transcriptional gene regulation (256). The synthesis and maturation of miRNA is divided into two stages, namely the formation of primary miRNA and precursor miRNA (257). The genes encoding miRNAs are transcribed into primary miRNAs in the nucleus under the action of RNA polymerase II. It is then processed by ribonucleases (Drosha and Dgcr8) to form a stem-loop precursor miRNA with a length of about 70 nucleotides (258). At this time, the precursor miRNA in the nucleus is transported to the cytoplasm by nucleocytoplasmic transporter, and processed by Dicer enzyme to mature miRNA product (259). The mature miRNA product is loaded into the RNA-induced silencing complex, binds to the Argonaute (Ago) 1–4 protein family to form a silencing complex, and guides the target messenger RNA (260). It plays a regulatory role by regulating gene expression through translational repression and degradation of target messenger RNAs (261, 262).

The clinical ischemic stroke usually has acute onset. Focal ischemia in the brain forms a complex cascade reaction (cerebral ischemia and hypoxia → energy metabolism disorder → release of excitatory neurotransmitters → free radical reaction → excessive calcium influx → apoptosis), which can be called “ischemic waterfall” (263–265). Studies have shown that miRNAs can be used to treat specific diseases. One of the advantages of miRNA therapy is that miRNA mimics and inhibitors can be artificially synthesized to alter the level of endogenous miRNAs, which have been used in the treatment of cancer and hepatitis C (266). Studies have shown that miRNAs are widely distributed, act in the posterior circulation and brain tissue of ischemic stroke (267), and participate in some pathological processes of the central nervous system. Alzheimer’s disease and multiple sclerosis are also associated with specific changes in miRNAs, indicating that miRNAs can be used as clinical biomarkers to obtain “liquid biopsies” derived from peripheral blood (17). miRNAs and target genes are involved in a variety of pathophysiological processes related to ischemic stroke, such as atherosclerosis, neurovascular regeneration, brain edema, inflammation, and apoptosis. This suggests that miRNA can be a biomarker for early diagnosis of ischemic stroke and an effective therapeutic target (268, 269). Studies have shown that miR-21 can be used as a biomarker and therapeutic target in different types of strok. Wang et al. (2018c) found that miR-3473b induced stroke neuritis by targeting microglial inhibitory factor 3, which indirectly contributed to the occurrence of stroke (270). Chua and Tang (2019) found that miR-34a affects mitochondrial activity, providing a new therapeutic strategy for targeting miR-34a in the treatment of stroke, Alzheimer’s disease and other cerebrovascular and neurodegenerative diseases (271).

### 5.1 miRNAs in whole blood, serum, plasma, cerebrospinal fluid as diagnostic biomarkers in patients with stroke

The current primary tissue-derived biomarkers for stroke patients are miRNAs analyzed from human whole blood, cerebrospinal fluid (CSF), serum, and plasma (272). Different patterns of miRNA expression at different time points may indicate their functions in different stages of ischemic stroke (273). Different miRNAs have been considered as diagnostic markers in patients with ischemic stroke using different samples such as blood, cerebrospinal fluid, plasma and serum. Blood miRNAs are the main source of biomarkers, and miRNAs enter the blood circulation through the brain interstitial fluid through the blood-brain barrier (274). Some brain-specific miRNAs do not cross the blood-brain barrier, whereas miRNAs in the peripheral circulation may be secreted by blood cells in response to more distal brain injury. Therefore, the miRNA profiles in ischemic brain and peripheral blood may be different.

The summary of miRNAs changes in whole blood, serum, plasma, cerebrospinal fluid as diagnostic biomarkers in patients with stroke were shown in **Table 2**.

### 5.2 miRNAs as prognostic biomarkers in stroke patients

Evidence from animal model studies supports that blood and brain miR-210 levels are associated with prognosis after cerebral infarction. found that blood miR-210 was associated with clinical findings in acute ischemic stroke involving 112 patients and 60 controls. MiR-210 levels were significantly elevated in stroke patients with favorable prognosis and controls compared with patients with poor prognosis. Thus, blood miR-210 was suggested as a novel sensitive biomarker for prognosis of acute cerebral ischemia in humans (275). miR-150-5p regulates not only pro-inflammatory cytokines but also vascular integrity. Scherrer et al. measured miR-150-5p levels within 72 hours of symptom onset in 329 ischemic stroke patients. They found that lower miR-150-5p levels were independently associated with mortality, and miR-150-5p emerged as a novel prognostic biomarker for ischemic stroke. This finding improves risk classification in patients with ischemic stroke beyond established risk factors (77). Edward et al. (2018) conducted a study to determine whether miRNAs differ in plasma from patients with different stroke recovery profiles. Twenty-seven ischemic stroke patients with mild to moderate upper extremity injury participated in the study, and plasma was collected 19 days after stroke. Six miRNAs, including miR-371-3p, miR-524, miR-520g, miR-1255A, miR-453 and miR-583, were significantly expressed in the well-recovery and poor-recovery groups, while three miRNAs (miR-941, miR-449b and miR-581) were significantly decreased (79). These miRNAs have been reported to be involved in axon guidance and developmental biology. The miRNAs associated with prognosis are summarized in **Table 3**.

### 5.3 miRNAs involved in neuroimmune inflammatory processes after ischemic stroke

#### 5.3.1 miR-155

MiR-155 is an important regulator of neuroinflammation (276). In MCAO mice and OGD-treated BV2 cells, miR-155 can activate NF-κB signaling by regulating TLR4 and downregulating the expression of suppressor of cytokine signaling (SOCS)-1 and myeloid differentiation factor 88 (80). Pena-Philippides et al. (2016) found that tail vein injection of miR-155 inhibitor in MCAO mouse model up-regulated the expression of its target proteins SOCS-1, SH2-containing 5’-inositol phosphatase-1 and CCAAT/enhancer-binding protein-β. It also simultaneously reduces STAT3 phosphorylation and reduces the expression of pro-inflammatory cytokines involved in vascular inflammation, such as pro-inflammatory chemokine ligand 12 and chemokine ligand 3, thereby affecting the inflammatory process. This has a positive effect on the inflammatory response after stroke. The high expression of serum miR-155, tyrosine protein kinase 2/STAT-3 and TNF-α in patients with AIS can trigger the inflammatory response after stroke, and miR-155 can be used as a potential inflammatory marker of AIS (277).

#### 5.3.2 miR-377

In the MCAO rat model and in microglia exposed to OGD, the expression of miR-377 was significantly decreased, and the expression of EGR2 and VEGF was increased, suggesting that miR-377 may regulate the anti-inflammatory effect of EGR2 and the angiogenic effect of VEGF. Knockdown of miR-377 inhibited the activation of microglia and the release of pro-inflammatory cytokines, and attenuated the inflammatory response in brain tissue. Injection of miR-377 inhibitor into the lateral ventricle of MCAO rats can reduce the volume of cerebral infarction, inhibit the inflammatory response, and promote angiogenesis (81). Furthermore, inhibition of miR-377 promoted capillary-like tube formation and cell proliferation and migration in BMECs. Knockdown of miR-377 attenuated ischemic brain injury by inhibiting brain inflammation, suggesting that miR-377 may be a potential therapeutic target for ischemic stroke (Fan et al., 2018).

#### 5.3.3 miR-1202

Song et al. (2020) found that the expression of miR-1202 was down-regulated and the expression of Rab1a was up-regulated in OGD/R-induced HM cells. Rab1a can up-regulate the expression level of TLR4 on the surface of HM cells induced by OGD/R, and TLR4 can bind to ligands and promote the activation of NF-κB inflammatory signaling pathway. miR-1202 was determined to directly target Rab1a, so overexpression of miR1202 could inhibit the activation of TLR4/NF-κB inflammatory signaling pathway to exert neuroprotective effects. In conclusion, some miRNAs can target the upstream signaling protein expression of NF-κB, thereby inhibiting the NF-κB signaling pathway to reduce apoptosis and reduce the inflammatory response to play a neuroprotective role (82).

#### 5.3.4 miR-21-5p

Ge et al. (2016) found that miR-21-5p inhibited the inflammatory response by regulating the expression of inflammatory cytokines and the nuclear factor κB signaling pathway through an *in vitro* scratch injury model of rat brain microvascular endothelial cells. On the other hand, miR-21-5p inhibits apoptosis by regulating the expression of apoptotic factors and the protein kinase B signaling pathway. Meanwhile, *in vitro* experiments also confirmed that miR-21-5p can promote the activation of angiopoietin 1/tyrosine kinase receptor 2 pathway. This suggests that miR-21 - 5p affects the activity of NF-κB, protein kinase B and angiopoietin 1/tyrosine kinase receptor 2 signaling pathway by inhibiting inflammation and apoptosis, and promotes angiogenesis repair in brain tissue and reduces vascular barrier leakage. Angiogenesis in damaged brain tissue promotes the recovery of the local vasculature, thereby providing a critical neurovascular substrate for neural remodeling, which has important implications for neuronal recovery after traumatic brain injury. Therefore, miR-21-5p may also be a target for the treatment of the blood-brain barrier after brain injury (83).

#### 5.3.5 miRNA-20b

The study found that down-regulation of miRNA-20b expression can inhibit the nucleotide binding oligomerization domain like receptor protein 3 (NLRP3) signaling pathway, reduce the IL-1β, IL-18 levels, adenosine triphosphate and reactive oxygen species during cerebral ischemia, thereby reducing the inflammatory injury after ischemic stroke (84).

#### 5.3.6 miRNA-634

Chang et al. (2018) found that reducing the expression of miRNA-634 can promote the growth of cerebral infarction cells and inhibit the neuroinflammatory response and apoptosis of cerebral ischemia (278).

#### 5.3.7 miRNA-124

Liu et al. (2015) found that within 24 hours after the onset of ischemic stroke patients, serum miRNA-124 and miRNA-9 decreased, and they were negatively correlated with cerebral infarct volume and CRP. Inhibiting the expression of miRNA-124 and miRNA-9 can promote neuroinflammation and brain injury (85).

#### 5.3.8 miRNA-203

In cerebral ischemia injury, miRNA-203 inhibits the downstream NF-κβ signaling pathway and activates microglia through negative feedback regulation of the adaptor protein MyD88. This suggests that miRNA-203 helps to inhibit the inflammatory response of cerebral ischemia and reduce neuronal damage, which provides a new strategy for the treatment of post-cerebral ischemia (86).

#### 5.3.9 miRNA-93

The study found that the expression of miRNA-93 in the brain tissue of mice with cerebral ischemia-reperfusion injury was significantly down-regulated. It may inhibit the inflammatory response and apoptosis after cerebral ischemia-reperfusion injury by targeting the interleukin 1 receptor-associated kinase 4 (IRAK4) signaling pathway, and reduce the expression of inflammatory factors in mouse microglia (87).

#### 5.3.10 miRNA-145

Nurr1 is a member of the orphan nuclear receptor 4 family, and its high expression can significantly inhibit the expression of TNF-α in microglia and reduce neuronal inflammatory and cytotoxic responses induced by cerebral ischemia-reperfusion. Inhibiting the expression of miRNA-145-5p can promote the expression of Nurr1 during acute cerebral ischemia, which is helpful for the recovery of neurological function and reducing the size of cerebral infarction. Therefore, blocking the axial signaling of miRNA-145-5p-Nurr1-TNF-α in the acute phase may be an effective therapeutic method to alleviate neuronal injury in cerebral ischemia-reperfusion (88).

#### 5.3.11 miRNA-424

After middle cerebral artery occlusion, miR-424-overexpressing treatment reduced infarct size and cerebral edema both before and after treatment. Meanwhile, lentiviral overexpression of miR-424 inhibited neuronal apoptosis and microglial activation, including inhibition of ionized calcium-binding adaptor molecule-1 immunoreactivity and protein levels, and reduced tumor necrosis factor-α production. *In vitro* studies showed that miR-424 mimics caused G1 cell cycle arrest and inhibited BV2 microglial activity (279). Furthermore, the expression level of miR-424 was increased in lymphocytes and neutrophils after stroke, suggesting its diagnostic value in ischemic stroke. MiR-424 levels in neutrophils were negatively correlated with infarct volume. Lymphocyte miR-424 levels were negatively correlated with lymphocyte number and expression of the cyclin-dependent kinase CDK6. In addition, plasma TNF-α and IGF-1 levels were increased and decreased in stroke patients, respectively, and miR-424 levels in both lymphocytes and neutrophils were negatively correlated with plasma TNF-α, IL-10, or IGF-1 levels. In conclusion, miR-424 levels in peripheral immune cells have diagnostic potential for ischemic stroke (89).

#### 5.3.12 miRNA-181c

TLR is expressed by microglia and astrocytes and can activate the nuclear factor κB signaling pathway, thereby promoting the expression of pro-inflammatory genes, cytokines and adhesion molecules. At present, a variety of TLRs have been discovered, and the TLR4 signaling pathway has been found to be involved in the regulation of inflammatory damage after ischemia. TLR4 is a direct target of miR-181c in microglia, which can down-regulate the expression of TRL4. In addition, miR-181c can also inhibit the activation of nuclear factor κB and the production of pro-inflammatory factors (90). Ma et al. (2016) found that miR-181c-3p was downregulated in OGD-treated cortical neurons and exosomes derived from OGD-treated cortical neurons. Exosomes derived from OGD-treated cortical neurons reduced the expression of CXCL1 and inflammatory factors in astrocytes, and exosomes delivered miR-181c-3p to reduce CXCL1 expression in astrocytes. CXCL1 is a target gene of miR-181c-3p. The use of a miR-181c-3p mimic and delivery of siRNA against CXCL1 (si-CXCL1) was shown to suppress astrocyte inflammation by downregulating CXCL1. Furthermore, LPS/H2O2 treatment inhibited BV2 microglia proliferation without inducing apoptosis, whereas miR-181c decreased proliferation but increased apoptosis in these cells with or without LPS/H2O2 treatment. LPS/H 2 O 2 induces apoptosis in Neuro-2a cells co-cultured with BV2 cells, and this effect is enhanced by miR-181c. In the MCAO model, miR-181c agomir modestly increased infarct volume, significantly decreased microglial activation and B-cell lymphoma 2 expression, and increased levels of pro-apoptotic proteins in the ischemic brain (280).

#### 5.3.13 miR-367-3p

In the MCAO mouse model, miR-367-3p was significantly decreased in the brain tissue of persistent ischemia, and the expression of its target gene G protein-coupled receptor C family 5A was significantly increased. The content of miR-367 - 3p in neurons is high, so the use of miR-367 - 3p mimics may inhibit the expression of pro-inflammatory genes in neurons by increasing the level of miR-367 - 3p in neurons, thereby reducing the inflammatory response related to ischemic stroke (281).

#### 5.3.14 miR-195

In the MCAO mouse model, miR-367-3p was significantly decreased in the brain tissue of persistent ischemia, and the expression of its target gene G protein-coupled receptor C family 5A was significantly increased. The content of miR-367 - 3p in neurons is high, so the use of miR-367 - 3p mimics may inhibit the expression of pro-inflammatory genes in neurons by increasing the level of miR-367 - 3p in neurons, thereby reducing the inflammatory response related to ischemic stroke. Yang et al. (2018b) studied the plasma samples of 96 AIS patients and found that the expression of miR-195 was significantly down-regulated, and was significantly negatively correlated with the National Institutes of Health Stroke Scale score. The expression of miR-195 in the plasma of MCAO mice was decreased. Intracerebroventricular injection of miR-195 lentivirus could inhibit the transduction of inflammatory signals. miR-195 mainly mediates the occurrence of neuroinflammation through CX3C chemokine receptor 1 (282). Mao et al. (2020) showed that MicroRNA-195 prevents the polarization of hippocampal microglia/macrophages to the M1 phenotype induced by chronic cerebral hypoperfusion by regulating CX3CL1/CX3CR1 signaling. It is suggested that miR-195 may become a biomarker of ischemic stroke and a new target for regulating neuroinflammation. In addition, OGD significantly inhibited the cell viability and induced apoptosis of HUVECs. Meanwhile, OGD treatment significantly decreased the expression of miR-195 and enhanced the expression of NF-κB (91). Furthermore, miR-195 directly interacts with IKKα and represses its expression. Mechanistically, overexpression of miR-195 exhibited pro-proliferative and anti-apoptotic effects on OGD-treated HUVEC by targeting the IKKα-mediated NF-κB pathway. At the molecular level, by inhibiting the IKKα/NF-κB pathway, miR-195 suppressed the expression of the pro-apoptotic protein Bax and active caspase-3, but increased the expression of the anti-apoptotic protein Bcl-2 in HUVECs. This suggests that miR-195 has a protective effect on the biological behavior of HUVECs by inhibiting IKKα-induced NF-κB pathway, which may provide a new potential strategy for clinical treatment of ischemic stroke (283).

#### 5.3.15 miR-183

Xiang et al. (2019) found that the expression of miR-183 was decreased after CIRI in rats. After using miR-183 analogues to treat rats with I/R injury, they found that the expression of pro-inflammatory proteins (IL-1β, IL-6 and TNF-α), the neurological function score of rats and the volume percentage of cerebral infarction decreased, the activity of microglia increased, and the expression of NF-κB p65 and I-κBα decreased and increased, respectively. This suggests that miR-183 regulates the activation of microglia in CIRI by inhibiting the NF-κB signaling pathway to protect nerves (92).

#### 5.3.16 miR-210

Wang (2018) found that in patients with acute cerebral infarction, the expression level of serum miRNA-210 and the levels of serum inflammatory factors CRP, IL-6 and NGAL were increased, suggesting that miRNA-210 may play a role in promoting inflammatory response (93). Huang et al. found that miR-210-LNA can significantly reduce cerebral infarction and improve MCAO-induced behavioral deficits, and at the same time improve long-term behavioral recovery in rats. Inhibition of miR-210 significantly reduced the expression of proinflammatory cytokines (TNF-α, IL-1β and IL-6) and chemokines (CCL2 and CCL3). It is suggested that inhibition of miR-210 shows pro-inflammatory response and reduces brain damage in the acute phase of ischemic stroke, providing a new strategy for the molecular basis of new therapeutic strategies for the treatment of acute ischemic stroke with miR-210 inhibition (98). Huang et al. (2018b) found that miR-210-LNA can significantly reduce cerebral infarction and improve MCAO-induced behavioral deficits, and at the same time improve long-term behavioral recovery in rats. Inhibition of miR-210 significantly reduced the expression of proinflammatory cytokines (TNF-α, IL-1β and IL-6) and chemokines (CCL2 and CCL3). It is suggested that inhibition of miR-210 shows pro-inflammatory response and reduces brain damage in the acute phase of ischemic stroke, providing a new strategy for the molecular basis of new therapeutic strategies for the treatment of acute ischemic stroke with miR-210 inhibition (284).

#### 5.3.17 miR-217

Inhibition of miR-217 attenuated CIRI injury by upregulating SIRT1 and SIRT1/AMPK-α/NF-κB pathways (285). *In vitro* experiments showed that miR-217 promoted the accumulation of HDAC5 in the nucleus by targeting MEF2D, resulting in decreased expression of IL-10. Furthermore, miR-217 repressed the expression of NADH dehydrogenase subunit 6 (ND6) in a MEF2D-dependent manner. Overexpression of MEF2D could reverse OGD-induced ND6 downregulation and OGD-mediated neuronal apoptosis, and could also reduce OGD-induced increase in ROS generation. In summmary, the MEF2D-HDAC5/ND6 signaling pathway regulated by miR-217 is involved in oxidative stress and inflammation after cerebral ischemia (119).

#### 5.3.18 miR-26b-5p

In the central nervous system, CIRI can lead to morphological changes and enhanced activity of microglia, and M1 activity of microglia is associated with BBB expansion by CIRI. miR-26b-5p attenuated the TLR pathway by targeting CH25H and inhibited microglial M1 polarization, thereby attenuating CIRI nerve injury (94). In addition, MicroRNA-26b-5p attenuated cerebral ischemia-reperfusion injury in rats by down-regulating KLF10 expression and inhibiting the N-myc/PTEN axis, reducing apoptosis and inflammatory responses (286). Further studies revealed that knockdown of the gene GAS5 could improve apoptosis and inflammatory responses by modulating the miR-26b-5p/Smad1 axis in CIRI rats. Smad1 overexpression reversed the inhibitory effects of miR-26b-5p on CI/R-induced apoptosis and inflammatory responses in rats. Collectively, these results suggest that knockdown of GAS5 can improve apoptosis and inflammatory responses by modulating the miR-26b-5p/Smad1 axis in CI/R rats (287).

#### 5.3.19 Other miRNA

Cai et al. found that overexpression of miR-542-3p attenuated OGD-induced activation of apoptosis, reactive oxygen species, and inflammatory responses, confirming that MSC-derived exosomal miR-542-3p prevents ischemia-induced glioma by inhibiting TLR4. cytoplasmic inflammatory response (95). This suggests a possible therapeutic strategy for cerebral ischemic injury using exosome delivery of miR-542-3p. Dong et al. found that miRNA-22 could alleviate ischemic stroke-induced inflammation in rat models or *in vitro* models by inhibiting the p38 MAPK/NF-κB pathway (96). Zhang et al. found that human umbilical cord mesenchymal stem cell-derived exosomal miR-146a-5p reduced microglia-mediated neuroinflammation by inhibiting the IRAK1/TRAF6 signaling pathway after ischemic stroke (97). Huang et al. found that inhibition of microRNA-210 suppressed pro-inflammatory responses and reduced acute brain injury in mice with ischemic stroke (149). Li et al. found that microRNA-381-3p promoted angiogenesis and inhibited inflammation by inhibiting Cebpb and Map3k8, thereby preventing ischemic stroke (99). Zheng et al. found that microRNA-421-3p prevented inflammatory responses in cerebral ischemia/reperfusion injury by targeting m6A Reader YTHDF1 to inhibit p65 mRNA translation (100). Zhou et al. found that miR-19a/b-3p promotes inflammation during cerebral ischemia/reperfusion injury through the SIRT1/FoxO3/SPHK1 pathway (101). Zhang et al. found that MicroRNA-665-3p inhibited NF-κB signaling by targeting TRIM8, and attenuated microglial apoptosis and inflammatory responses induced by oxygen-glucose deprivation (102). Huang et al. found that elevated microRNA-135b-5p alleviated neuronal damage and inflammation in post-stroke cognitive impairment by targeting NR3C2 (103). Bao et al. found that MiR-5787 attenuated macrophage-mediated inflammation by targeting TLR4/NF-κB in ischemic cerebral infarction (104). Liu et al. found that elevation of c-myc or inhibition of miR-200b-5p improved neurological function, reduced inflammation and neuronal apoptosis, and attenuated brain histopathology and neuronal survival in MCAO mice (105). Enhancement of miR-200b-5p or knockdown of SIRT1 attenuated c-myc-induced protection against MCAO-induced brain injury in mice. Shi et al. found that MicroRNA-532-5p prevented apoptosis, reactive oxygen species (ROS), and inflammation in cerebral ischemia-reperfusion injury by directly targeting CXCL1 (106). Ye et al. found that serum exosomal microRNA-27-3p aggravated brain injury and inflammation in patients with acute cerebral infarction by targeting PPARγ (107). Tu et al. found that MiRNA-34c-5p protects against cerebral ischemia/reperfusion injury and is involved in anti-apoptotic and anti-inflammatory activities (108). Shan et al. found that miR-221 exerts a neuroprotective effect in ischemic stroke by inhibiting the pro-inflammatory response (109). Xie et al. found that upregulation of miR-874-3p inhibited CXCL12 expression, thereby promoting angiogenesis and suppressing the inflammatory response in ischemic stroke (110). Deng et al. found that miR-671-5p attenuated neuroinflammation by inhibiting NF-κB expression in an acute ischemic stroke model (26). Zha et al. found that remote ischemic preconditioning reduces infarct volume and improves neurological function in acute ischemic stroke in part through miR-153-5p/TLR4/p65/IkBa signaling pathway (111).

Liu et al. found that MiR-92b-3p regulates OGD/R-mediated apoptosis and inflammation by targeting TRAF3 in PC12 cells (112). Yang et al. found that inhibition of miR-15a/16-1 attenuated ischemic brain injury by upregulating anti-apoptotic proteins and inhibiting pro-inflammatory molecules, suggesting that miR-15a/16-1 is a new therapeutic target for ischemic stroke (113). Marcin et al. found that PPAR-β/δ receptor agonist GW0742 can activate PPAR-β/δ, which can activate the expression of miR-17-5p in PC12 cells and increase cell viability after OGD. This was accompanied by a decrease in the expression of TXNIP and a decrease in the secretion of IL-1β and TNF-α (114). Tian et al. found that the up-regulation of miR-216a exerts neuroprotective effects against ischemic injury by negatively regulating the apoptosis and inflammatory pathways involved in JAK2/STAT3 (115). Wang et al. found that MicroRNA-182-5p attenuated cerebral ischemia-reperfusion injury by targeting Toll-like receptor 4 (116). Zhao et al. found that exosomes from MSCs overexpressing microRNA-223-3p attenuated cerebral ischemia by inhibiting microglial M1 polarization-mediated inflammation (117). Yang et al. found that MiR-582-5p attenuates neonatal hypoxic-ischemic encephalopathy by targeting high mobility group box 1 (HMGB1) by inhibiting neuroinflammation and oxidative stress (118). Shi et al. found that the MEF2D-HDAC5/ND6 signaling pathway regulated by miR-217 is involved in oxidative stress and inflammatory responses after cerebral ischemia (119). Chen et al. found that MicroRNA-193b-3p alleviated focal cerebral ischemia and reperfusion injury in rats by inhibiting the expression of 5-lipoxygenase (120). Zhou et al. found that MiR-199a regulates autophagy and inflammation in rats with cerebral infarction by regulating mTOR expression (121). Fang et al. found that MiR-544 inhibited inflammatory response and apoptosis after cerebral ischemia-reperfusion by targeting IRAK4 (122). Yu et al. found that endothelial cell-derived exosomal miR-199a-5p reduced neuronal apoptosis and inflammation by inhibiting endoplasmic reticulum stress (123). Ni et al. found that MiR-let-7c-5p protects against cerebral ischemic injury through a mechanism involving inhibition of microglial activation (124). Song et al. found that MiR-1202 exerted neuroprotective effects on OGD/R-induced HM cell inflammation by negatively regulating Rab1a involved in the TLR4/NF-κB signaling pathway (82). Liu et al. found that MiR-665 was involved in the protective effect of dexmedetomidine on ischemic stroke through the ROCK2/NF-κB axis (125). Wang et al. found that MiR-139 prevented oxygen-glucose deprivation/reoxygenation (OGD/R)-induced nerve damage by targeting c-Jun to inhibit NLRP3 inflammasome activation (126). Dong et al. found a potential role of microRNA-7 in the anti-neuroinflammatory effect of nicorandil in oxygen-glucose deprivation-induced astrocytes (127). Wu et al. found that miR-124-5p/NOX2 Axis regulates ROS production and inflammatory microenvironment to prevent brain I/R injury (128). Ma et al. found that resveratrol promoted M2 polarization of microglia and reduced neuroinflammation after cerebral ischemia by inhibiting miR-155 (129). Resveratrol promotes M2 polarization of microglia and reduces neuroinflammation after cerebral ischemia by inhibiting miR-155. Li et al. found that adipose-derived mesenchymal stem cells attenuated ischemic brain injury in rats by regulating miR-21-3p/MAT2B signaling (130). Xu et al. found that MiR-1906, a novel Toll-like receptor 4 modulator, ameliorated ischemic injury after experimental stroke in mice (288). Liu et al. found that Electroacupuncture inhibits inflammatory damage after ischemic stroke by targeting miR-9-mediated NF-κB signaling pathway (131). Song et al. found that sevoflurane protected mice from neuronal apoptosis and inflammation in ischemic brain injury by modulating the microRNA-203-3p/HDAC4/Bcl-2 axis (132). Song et al. found that cortical neuron-derived exosomal MicroRNA-181c-3p inhibited neuroinflammation by downregulating CXCL1 in astrocytes in a rat model of ischemic brain injury (133). The mechanisms of miRNAs in cerebral ischemia/ischemia-reperfusion injury were summarized in **Table 4** and shown in **Figure 5**. Taking the Wnt signaling pathway as an example, **Figure 4** shows the action mode of miRNAs in the Wnt signaling pathway.

## 6 Mechanisms of circRNA involved in neuroinflammation and immune regulation

### 6.1 The discovery and formation mechanism of circRNA

CircRNA is a kind of non-coding RNA. It was first reported in the study of viroids by Hsu and Coca-Prados in the late 1970s. Later, the existence of circRNA was observed in Sendai virus, yeast, and Tetrahymena (289). With the application of high-throughput sequencing technology, a large number of studies have reported the expression of various circRNAs in different species such as Caenorhabditis elegans, Drosophila, mice and humans (290). Initially, circRNA was thought to be a transcriptional by-product of gene abnormality, splicing, and little attention was paid to circRNA. With the development of transcriptomics and bioinformatics technology, the study of human fibroblast transcriptome genes found that the production of circRNA is mainly formed by post-transcriptional splicing of 14.4% of protein-coding genes in the organism. This indicates that more than 1/8 of protein-coding genes in human fibroblasts can generate circRNAs through post-transcriptional cleavage (291). Current studies have shown that the functions of circRNAs mainly include: (1) The “sponge effect” of miRNAs (292). In addition to serving as miRNA “sponges”, circRNAs are also related to the storage, classification and localization of miRNAs (293); (2) cis-regulated parental gene expression (285, 294); (3) The protein-related roles of circRNAs include translating proteins as templates and binding with RBPs to exert biological functions. A large number of research data show that circRNAs play a key role in the central nervous system. For example, it is involved in the occurrence and development of neural electrical activity, synaptic plasticity, neurodegeneration and irreversible nerve damage (295). The specific mechanisms of circRNAs in the brain include: (1) circRNAs are up-regulated in stress injury, and their stable expression can act as “stress memory molecules”; (2) circRNA binds to RBP and acts as a hub for protein transport to axons or dendrites; (3) circRNAs can encode stored information or participate in information transfer between cells.

### 6.2 Changes in the expression of circRNAs profile after the onset of AIS

Mehta et al. established a tMCAO model in male C57BL6J mice, and detected the differential expression of 1320 circRNAs at 6, 12, and 24 h of reperfusion. Among them, 283 circRNAs were altered (>2-fold) at at least 1 reperfusion time point, and 16 circRNAs were altered at all 3 reperfusion time points. Quantitative real-time polymerase chain reaction (qRT-PCR) validation results showed that the expressions of circ_008018, circ_015350 and circ_016128 were up-regulated, and the expressions of circ_011137, circ_001729 and circ_006696 were down-regulated (150). The main biological and molecular functions involved in these circRNAs are biological regulation, metabolic processes, cellular communication, and binding to proteins, ions, and nucleic acids. Liu et al. found that 1027 circRNAs were significantly changed in ischemic brain tissue after 45 min of tMCAO and 48 h of reperfusion in mice (296). The results of qRT-PCR verification showed that the circRNAs numbered mmu-circRNA-40001, mmu-circRNA-013120 and mmu-circRNA-25329 were differentially expressed, which mainly regulate cell proliferation and survival by participating in the Rapl pathway and the Hippo pathway. Ostolaza et al. conducted a comprehensive circRNA expression profiling analysis on 30 AIS patients with different etiologies (152): they compared large artery atherosclerotic stroke and cardioembolism patients and found that 219 circRNAs were differentially expressed, of which 60 cases of up-regulated circRNAs showed more than 4-fold changes (152); they compared large-artery atherosclerotic stroke and stroke of unknown etiology and found that 226 circRNAs were differentially expressed, of which 87 were up-regulated, 139 were down-regulated, and only one circRNA showed a more than 4-fold change in expression (152); they compared cardioembolism and stroke of unknown etiology and found that only 8 circRNAs were up-regulated and 9 circRNAs were down-regulated (152). The results of qRT-PCR validation showed that only the HSA_CircRNA 102488 originated from the UBA52 ribosomal protein fusion product 1 (UBA52) gene located on chromosome 19 was statistically significant between different etiological subtypes, and Its RBP sites are clustered around AGO2 and FUS proteins. Functional analysis showed that the differentially expressed circRNAs mainly interacted with a group of AIS-related miRNAs. Its functions include fatty acid biosynthesis, lysine degradation, arrhythmogenic right ventricular cardiomyopathy or hypertrophic cardiomyopathy (152).

Lu et al. analyzed the expression levels of circRNAs in the blood of tMCAO model mice and validated the selected circRNAs in AIS patients. The results showed that 128, 198 and 789 circRNAs were significantly altered at 5 min, 3 h and 24 h after cerebral ischemia, respectively. Their target genes were related to Hippo signaling pathway, extracellular matrix-receptor interaction and fatty acid metabolism, respectively (153). Finally, it was found that circBBS2 and circPHKA2 were differentially expressed in the blood of AIS patients. Li et al. found that a total of 2 659 circRNAs were significantly altered in the ipsilateral thalamus of adult male C57BL/6J mice in the permanent distal MCAO model compared with the sham-operated group (154). Among them, 73 circRNAs were significantly changed at 2 time points after modeling (154). GO and KEGG analysis showed that circRNAs play an important role in secondary thalamic neurodegeneration and remodeling after focal cortical infarction, suggesting that circRNAs may be therapeutic targets to alleviate secondary distal neurodegeneration after stroke. Dong et al. (155) used high-throughput sequencing (HTS) technology to compare the expression of circRNAs in peripheral blood mononuclear cells (PBMCs) of 5 patients with AIS and 5 healthy subjects. They found that 521 circRNAs were differentially expressed in AIS patients compared with healthy subjects, of which 373 circRNAs were up-regulated and 148 circRNAs were down-regulated. The results of qRT-PCR validation showed that 8 circRNAs contained sites that bind to multiple miRNAs. GO and KEGG analysis showed that abnormally expressed circRNAs were involved in many pathophysiological processes of AIS, especially inflammatory and immune processes. Lin et al. studied the differences in circRNAS expression between OGD/R HT22 cells and normal control cells. They found that 15 circRNAs were significantly altered, of which 3 were significantly up-regulated and 12 were significantly down-regulated (156). The results of qRT-PCR validation showed that the up-regulated MMU-circRNA-015947 could interact with miRNAs to enhance the expression of target genes, and participate in the process of ischemia-reperfusion injury by participating in apoptosis metabolism and immune-related pathways. The study by Duan et al. using the rat MCAO model showed that 87 differentially expressed circRNAs showed more than 2-fold changes (157). qRT-PCR validation results showed that the significantly up-regulated circRNAs included circRNA 17737, circRNA 8828 and circRNA 14479, while circRNA1059, circRNA9967 and circRNA6952 were significantly down-regulated. Overall, these studies suggest that circRNAs have potential applications in the diagnosis, treatment, and prediction of AIS.

### 6.3 The role of circRNAs in AIS

At present, more and more studies have shown that circRNAs play multiple roles in the occurrence and recovery of AIS, but their value in AIS prediction still needs to be further clarified. The potential functions and interactions of circRNAs in the pathophysiological mechanisms of AIS mainly involve ischemic brain injury, protection of the blood-brain barrier, inhibition of apoptosis, and neuroprotection and neuroinflammation.

#### 6.3.1 circHECTDI, circTLK1 and circ-camk4 play key roles in the process of cerebral ischemia-reperfusion injury

Using circRNAs microarray analysis, Han et al. found that circHECTDI levels were significantly increased in ischemic brain tissue from tMCAO model mice and in plasma samples from AIS patients. Inhibition of circhetdl expression significantly reduced infarct volume, alleviated neurological deficits and improved astrocyte activation. This suggests that circHECTDI and its coupling mechanism are involved in the occurrence of cerebral ischemia, and it is expected to serve as a new stroke biomarker and therapeutic target (134). Wu et al. found that the level of circTLK1 in the brain tissue of the focal ischemia-reperfusion mouse model and in the plasma of AIS patients was significantly increased, and knockout of circTLK1 could significantly reduce the infarct volume, reduce neuronal damage and improve neurological deficits (135). Zhang et al. found that the expression level of circ-camk4 in OGD/R neurons was significantly increased, and overexpression of hsa-circ-camk4 could significantly increase the cell death rate after OGD/R, suggesting that circ-camk4 may play a key role in the process of cerebral ischemia-reperfusion injury (297). Yang B et al. found that in cerebral infarction, circTTC3 regulates CIR injury and neural stem cells (NSCs) through the miR-372-3p/TLR4 axis (298).

#### 6.3.2 circRNA DLGAP4, circ_0072309, circ_016719 and circCCDC9 protect blood-brain barrier and inhibit apoptosis after cerebral infarction

Bai et al. showed that circRNA DLGAP4 (circDLGAP4) can act as an endogenous miR-143 sponge to inhibit miR-143 activity. Plasma circDLGAP4 levels were significantly reduced in AIS patients and in a mouse model of cerebral ischemia (136). The results of *in vivo* experiments showed that up-regulation of circDLGAP4 expression can significantly reduce neurological deficits, reduce infarct volume and reduce blood-brain barrier damage. The results of *in vitro* experiments showed that overexpressed circDLGAP4 could alleviate the protein downtrend caused by OGD/R and reduce the expression of mesenchymal cell markers, thereby inhibiting endothelial-mesenchymal transition and reducing blood-brain barrier damage, suggesting that circDLGAP4 may serve as a novel therapeutic target for acute cerebrovascular injury. Zhao et al. found that the expression level of circ 0072309 in the serum of IS patients and ischemic brain tissue of MCAO model mice was significantly decreased, while the content of miR-100 was significantly increased, suggesting that the circ_0072309-miR-100-mTOR regulatory axis can alleviate ischemic brain injury and is expected to serve as a potential therapeutic target for AIS (299). Tang et al. found that circ016719 directly targets miR-29c, thereby regulating the expression and function of Map2k6 and promoting apoptosis (299). Wu et al. found that the expression of circCCDC9 in the brain of tMCAO model mice was significantly reduced, and overexpression of circCCDC9 could protect the blood-brain barrier and reduce apoptosis by inhibiting the Notchl signaling pathway (138). Therefore, circCCDC9 is expected to serve as a potential target for cerebrovascular protection in the acute phase of AIS. Chen et al. found that the level of circUCK2 was significantly reduced in the brain tissue of a mouse model of focal cerebral ischemia-reperfusion (300). Up-regulated circUCK2 levels significantly reduced infarct volume, attenuated neuronal damage, and ameliorated neurological deficits. circUCK2 reduces oxygen-glucose deprivation (OGD)-induced apoptosis by regulating transforming growth factor beta (TGF-β)/mothers against decapentaplegic homolog 3 (Smad3) signaling. Furthermore, circUCK2 functions as an endogenous miR-125b-5p sponge, inhibiting miR-125b-5p activity, resulting in increased growth differentiation factor 11 (GDF11) expression followed by amelioration of neuronal damage. These findings suggest that the circUCK2/miR-125b-5p/GDF11 axis is an important signaling pathway during ischemic stroke. Therefore, circRNA circUCK2 can be used as a potential target for the treatment of patients with AIS (300).

#### 6.3.3 circRNA SCMHI, circHIPK2, circ-HECTDI, circRNA SHOC2 and circ_0078299 are involved in neuroprotection after cerebral infarction

Yang et al. found that the levels of circRNA SCMHI in plasma and peri-infarct cortex of AIS patients and cerebral infarction model mice were significantly reduced, and treatment with exogenous circSCMHI could significantly improve the functional recovery of cerebral ischemia model mice and monkeys. The mechanism is related to enhancing neuronal plasticity and inhibiting glial cell activation and peripheral immune cell infiltration (301).

Wang et al. found that the use of neural stem cells that silence Si-circHIPK2 expression can improve neuronal plasticity in ischemic brain tissue, confer long-term neuroprotection, and significantly improve neurological function (141). Dai et al. found that in OGD-induced HT22 cells and MCAO model mice, the expressions of circRNA HECTDI (circHECTDI) and tumor necrosis factor receptor-associated factor 3 (TRAF3) were significantly up-regulated, and the expression of miR-133b was down-regulated (142). TRAF3 is one of the targets of miR-133b. Circ-HECTDI knockdown attenuated OGD-induced neuronal death, while reducing cerebral infarct volume and neuronal apoptosis in MCAO mice. Chen et al. found that circRNA SHOC2 (circSHOC2) in ischemic preconditioned astrocyte exosomes inhibited neuronal apoptosis and alleviated neuronal damage by regulating autophagy and acting on the miR-7670-3p/SIRT1 axis (143), which may contribute to the development of AIS treatment strategies. Silva et al. found that hsa_circ_0078299 and FXN may be new biomarkers of AIS, which can achieve neuroprotection and brain recovery by participating in some well-known stroke-related biological processes (144).

#### 6.3.4 circDLGAP4, circHECTD1, circDLGAP4, and circSCMH1 are involved in neuroinflammation after cerebral infarction

Astrocytes is the neuroinflammatory regulator, and activation of astrocytes in the brain can aggravate the inflammatory response and neuronal damage, so inhibiting astrocyte activation can increase the level of circHECTD1 in the brain tissue of tMCAO mice. significantly increased, and the expression of miR-142 decreased. *In vitro* studies found that miR-142 and circHECTD1 co-transduced astrocytes, and circHECTD1-induced astrocyte activation was inhibited by overexpressed miR-142 (134). It has been found that the expression of circHECTD1 in peripheral blood of patients with AIS is increased, and the expression of circHECTD1 is positively correlated with the National Institutes of Health Stroke Scale score, CRP and pro-inflammatory cytokines. This suggests that circHECTD1 may serve as a biomarker for the risk, severity, inflammation and recurrence of AIS (146).

The blood-brain barrier is an important structure to maintain the homeostasis of the central nervous system, and the destruction of the blood-brain barrier can lead to peripheral inflammatory infiltration, amplify neuroinflammatory responses, and lead to neuronal damage. circDLGAP4 expression was downregulated in tMCAO mice and AIS patient plasma. circDLGAP4 can act as an endogenous miR-143 sponge, interfere with the expression of Hect domain E3 ubiquitin protein ligase 1, inhibit endothelial cell-mesenchymal transition, and destroy the integrity of the blood-brain barrier (136). In addition, extracellular vesicles can penetrate the blood-brain barrier and carry proteins, lipids, and nucleic acids. The level of circSCMH1 was decreased in the plasma of AIS patients and in the brain tissue of light-embolized stroke mice. Constructing extracellular vesicles containing circSCMH1 and injecting them into mice *via* tail vein can reduce glial cell activation and peripheral immune cell infiltration, which is beneficial to promote functional recovery after stroke (140). Zhu et al. found that the expression level of circDLGAP4 in PBMCs of patients with AIS was negatively correlated with miR-143, and positively correlated with the National Institutes of Health Stroke Scale score and the levels of various pro-inflammatory cytokines. Therefore, circDLGAP4 is considered as a new biomarker for AIS diagnosis and disease monitoring (145). Peng et al. found that the expression level of circRNA HECTDI was associated with higher AIS disease risk, severity, inflammatory response and recurrence risk (146).

### 6.4 Potential role of circRNAs in predicting AIS

The development of simple, non-invasive and economical tools for early screening of vulnerable plaques is of great significance for the early prediction of AIS. Bazan et al. found that the serum miR-221 level in patients with symptomatic carotid stenosis in the acute phase was significantly lower than that in the asymptomatic group, while the circR-284 level was significantly higher than that in the asymptomatic group. The serum circR-284/miR-221 ratio may be used as a diagnostic biomarker for carotid plaque rupture and AIS. Identifying patients at high risk for stroke-associated infection (SAI) and administering prophylactic antibiotic therapy is critical for patients with AIS (147). Zuo et al. found that the level of circFUNDC1 was significantly increased in SAI patients, and the receiver operating characteristic curve confirmed that circFUNDCI had a significant predictive value for SAI. In addition, a positive correlation between circFUNDCI levels and neutrophil counts was also observed. Therefore, circFUNDCI can be used to construct a risk prediction model to predict SAI (302). Li et al. found that the circular RNA hsa_circ_0001599 can be used as a novel biomarker for large atherosclerotic stroke (159). The results of Liu et al. suggest that circ-STAT3 may be a novel biomarker for predicting functional outcomes after stroke and an important factor in IS recovery (303). According to Chen et al., the expression of hsa-circ-0141720 was most increased in the serum of ACI patients (147). Further studies showed that the high expression of hsa-circ-0141720 was closely related to NIHSS score, infarct volume, IL-6 and hs-CRP. The high expression of hsa-circ-0141720 in the serum of ACI patients is related to the severity of the disease and can be used as a new serological indicator for the diagnosis of ACI. The mechanisms of circRNAs in cerebral ischemia were summarized in **Tables 5**, **6** and shown in **Figure 5**.

## 7 Epilogue

At present, the use of ncRNAs as biomarkers for stroke diagnosis and prognosis still faces challenges, especially the research on circRNAs is still in its infancy. Several studies have shown that the expression of non-coding RNAs changes significantly after the onset of AIS, and ncRNAs are closely related to stroke severity and inflammatory response, and play an important role in stroke diagnosis, prognosis and treatment. However, there are currently no biomarkers for predicting stroke onset. With the development of novel sequencing technologies and bioinformatics methods, a large number of circRNAs have been discovered in different kinds of organisms and tissues. These findings expand our understanding of the classification and function of RNAs. The relationship between ncRNAs and the occurrence, development and prognosis of stroke and the specific mechanism will be gradually elucidated, providing new ideas for the early diagnosis and treatment of stroke targeting ncRNAs.

## 8 Conclusion and prospect

In this review, it is reviewed that ncRNA can affect the pathological process of IS in ischemic stroke, including neuroimmune inflammation and other processes, and can affect the functions of brain neurons, glial cells, cerebral microvascular endothelial cells, macrophages and other cells in patients with IS (see **Figure 5**). This suggests that ncRNAs play an important role in the pathogenesis of IS, which may become a potential target for the treatment of IS in the future. Although the potential of ncRNAs in AIS therapy may lead to exciting future directions, many challenges remain. These challenges are similar to those associated with ncRNA-based cancer therapy, including rapid degradation and clearance, poor cellular uptake, off-target effects, and immunogenicity (304). Off-target effects cannot be ignored, as they are the main cause of side effects and poor efficacy in miRNA-based therapies (305, 306). However, to date, there are no studies on off-target effects of miRNA-based therapy in AIS. To address this conundrum, comprehensive research is required in the future. Furthermore, miRNAs have been extensively studied so far, while the roles of lncRNAs and circRNAs in ischemic stroke remain largely unknown. Further studies may uncover these ncRNAs, which may also help us better understand the mechanism of cerebral ischemic injury. Most existing studies linking ncRNAs to ischemic stroke have been performed using rodent models of MCAO and *in vitro* OGD/R models. These experimental models will continue to help define the role of ncRNAs in AIS, whether through the use of genetic manipulation or through the use of ncRNA antagomirs or agomirs. Despite the rapid progress in miRNA biopharmaceutical research, ncRNA-based therapies have not yet been used in clinical practice. miRNA mimics and inhibitors have been shown to penetrate the BBB and prevent cerebral ischemic injury in animal models of AIS. Exosomes, liposomes, and lentiviruses may be used to carry ncRNA-based drugs and bring them to cerebral infarction areas (307–309).

At present, many studies have shown that the CRISPR/Cas9 system is the best choice for ncRNA-related genome editing or regulation (310–317). As one of the main ncRNAs, miRNAs have been extensively studied in CRISPR/Cas9 related fields. Chang et al. demonstrated that changes in the structure of primary miRNAs produced by the CRISPR/Cas9 system can lead to downregulation of mature miRNAs *in vivo* and *in vitro* (310). Meanwhile, this study also shows that the correct design of sgRNA in CRISPR/Cas9 can significantly minimize off-target effects of miRNAs with highly conserved sequences in the same family. Zhou et al. successfully knocked out miRNA-3188 in hepatoma cell lines using the CRISPR/Cas9 system, and found that miR-NA-3188KO could effectively inhibit cell growth, invasion and migration, and inhibit xenotransplantation in nude mice (318). tumor growth. In addition to editing or regulating the above-mentioned miRNAs, the CRISPR/Cas9 system has also shown wide application in various organisms for other miRNAs such as miRNA-137, miRNA-93, miRNA-309, miRNA-126a/b, etc. (312–316). In addition to miRNAs, another ncRNA that mainly regulates various biological processes, namely lncRNAs, has been successfully regulated by the CRISPR/Cas9 system. Studies have shown that a brain-specific long noncoding RNA called Fos downstream transcript (FosDT) is rapidly induced in rodent brains after focal ischemia (319). Using FosDT knockout rats, Suresh et al. assessed the role of FosDT in brain injury after stroke (320). They used CRISPR-Cas9 genome editing in the Sprague-Dawley context to generate FosDT knockout rats. Male and female FosDT-/- and FosDT+/+ cohorts suffered from transient middle cerebral artery occlusion. FosDT-/- rats did not show any abnormality in growth and development, fertility, brain cell structure and cerebrovascular system. However, both sexes FosDT-/- rats exhibited enhanced sensorimotor recovery and reduced brain damage when subjected to transient focal ischemia. RNA-sequencing analysis indicated that improved functional outcomes after stroke in FosDT-/- rats were in part related to reduced induction of inflammatory genes, reduced apoptosis, mitochondrial dysfunction, and oxidative stress. It can be seen that the CRISPR/Cas9 system can regulate the expression of lncRNA and provide a new method for clinical treatment of cerebral infarction/cerebral ischemia-reperfusion injury. Deletion or insertion of tiny genes may not necessarily result in loss of function of a non-coding gene, which may be one of the limitations of applying the CRISPR/Cas9 system to non-coding genes. To overcome this, Ho et al. used homologous recombination technology to integrate marker genes into the genome, and successfully knocked out UCA1, IncRNA-21 and AK023948 in HTC-116 and MCF-7 cells through the CRISPR/Cas9 system (321). In the study of SHECHNER et al., researchers developed the CRISPR-Display (CRISP-Disp) method, a targeted localization method using Cas9 to deploy macromolecular RNA vectors to DNA loci (60). They found that functional RNA domains of at least 4.8 bp in length can be inserted into multiple sites in CRISPR gRNAs, allowing the construction of Cas9 complexes with native lncRNAs. This approach may open up a new avenue for lncRNA research in the field of cerebral infarction/cerebral ischemia-reperfusion injury.

In conclusion, ncRNAs-derived therapy may be a novel adjuvant therapy strategy to improve the prognosis of IS after intravenous thrombolysis and endovascular mechanical reperfusion. However, in order to further translate these research results on ncRNA in the field of IS and carry out clinical research, some related work needs to be done: first, the safety of ncRNA needs to be clarified. In particular, the main method for the treatment of IS is intravenous thrombolysis with recombinant tissue plasminogen activator (rt-PA). In the future, the further development of this ncRNA to treat IS requires a benefit/risk assessment analysis. In addition, future targets need to design dosage forms suitable for human use. *In vivo* and *in vitro* models of cerebral ischemia/reperfusion: the intervention of ncRNA is carried out by constructing plasmids or lentiviral vectors, and when applying to humans, it is necessary to design a dosage form suitable for human use. Finally, it is necessary to design a mode of administration suitable for human use. In order to rapidly administer the constructed plasmid or virus carrying ncRNA to the brain tissue of experimental animals, the plasmid is generally injected into the lateral ventricle. For those patients who underwent mechanical thrombectomy, plasmids could be administered to the lesion along the surgical pathway when the mechanical thrombectomy was completed, while for patients who did not undergo mechanical thrombectomy, appropriate administration methods should be considered.

In summary, there have been many studies on the role of ncRNAs on IS, and these research results will be further transformed. To carry out clinical research, it is also necessary to clarify the safety of LncRNAs. In addition, it is necessary to design the dosage form and mode of administration suitable for human use.

## Author contributions

KY, LZ, AG, XY contributed equally to this work. All authors contributed to the article and approved the submitted version.

## Funding

This work is supported by the National Natural Science Foundation of China (81774174), the National Key Research and Development Project of China (No. 2018YFC1704904), Hunan Provincial Natural Science Foundation of China (2020JJ5424), Hunan University of Chinese Medicine “Double First-Class” Discipline Open Fund Project of Integrated Traditional Chinese and Western Medicine (2020ZXYJH08), Hunan Provincial Department of Education Youth Fund Project (21B0386).

## Conflict of interest

The authors declare that the research was conducted in the absence of any commercial or financial relationships that could be construed as a potential conflict of interest.

## Publisher’s note

All claims expressed in this article are solely those of the authors and do not necessarily represent those of their affiliated organizations, or those of the publisher, the editors and the reviewers. Any product that may be evaluated in this article, or claim that may be made by its manufacturer, is not guaranteed or endorsed by the publisher.
