# Supplementary material for: A systematic review of the research progress of non-coding RNA in neuroinflammation and immune regulation in cerebral infarction/ischemia-reperfusion injury
Source: Front Immunol. 2022 Oct 7;13:930171. doi: 10.3389/fimmu.2022.930171 (PMC9585453; doi:10.3389/fimmu.2022.930171)
Supplement: Supplementary file 1 [file Table_1.docx]

**Table S1.** Search Strategies for Pubmed and Embase

| **PubMed** | ((Untranslated RNA OR npcRNA OR RNA, Nontranslated OR Nontranslated RNA OR RNA, Non-Peptide-Coding OR Non-Peptide-Coding RNA OR RNA, Non Peptide Coding OR RNA, Non-Protein-Coding OR Non-Protein-Coding RNA OR RNA, Non Protein Coding OR RNA, Noncoding OR Noncoding RNA OR RNA, Non-Coding OR Non-Coding RNA OR RNA, Non Coding) OR (Noncoding RNA, Long OR lncRNA OR Long ncRNA OR ncRNA, Long OR RNA, Long Non-Translated OR Long Non-Translated RNA OR Non-Translated RNA, Long OR RNA, Long Non Translated OR Long Non-Coding RNA OR Long Non Coding RNA OR Non-Coding RNA, Long OR RNA, Long Non-Coding OR Long Non-Protein-Coding RNA OR Long Non Protein Coding RNA OR Non-Protein-Coding RNA, Long OR RNA, Long Non-Protein-Coding OR Long Noncoding RNA OR RNA, Long Untranslated OR Long Untranslated RNA OR Untranslated RNA, Long OR Long ncRNAs OR ncRNAs, Long OR Long Intergenic Non-Protein Coding RNA OR Long Intergenic Non Protein Coding RNA OR LincRNAs OR LINC RNA OR LincRNA) OR (MicroRNA OR miRNAs OR Micro RNA OR RNA, Micro OR miRNA OR Primary MicroRNA OR MicroRNA, Primary OR Primary miRNA OR miRNA, Primary OR pri-miRNA OR pri miRNA OR RNA, Small Temporal OR Temporal RNA, Small OR stRNA OR Small Temporal RNA OR pre-miRNA OR pre miRNA) OR (circRNAs OR Closed Circular RNA OR Circular RNA, Closed OR RNA, Closed Circular OR Circular RNA OR Circular RNAs OR RNAs, Circular OR circRNA OR Circular Intronic RNA OR Intronic RNA, Circular OR RNA, Circular Intronic OR ciRNA)  AND  (Cerebral Infarctions OR Infarctions, Cerebral OR Infarction, Cerebral OR Cerebral Infarct OR Cerebral Infarcts OR Infarct, Cerebral OR Infarcts, Cerebral OR Cerebral Infarction, Left Hemisphere OR Left Hemisphere, Infarction, Cerebral OR Infarction, Left Hemisphere, Cerebral OR Left Hemisphere, Cerebral Infarction OR Cerebral, Left Hemisphere, Infarction OR Infarction, Cerebral, Left Hemisphere OR Subcortical Infarction OR Infarction, Subcortical OR Infarctions, Subcortical OR Subcortical Infarctions OR Posterior Choroidal Artery Infarction OR Anterior Choroidal Artery Infarction OR Cerebral Infarction, Right Hemisphere OR Right Hemisphere, Cerebral Infarction OR Infarction, Right Hemisphere, Cerebral OR Right Hemisphere, Infarction, Cerebral OR Cerebral, Right Hemisphere, Infarction OR Infarction, Cerebral, Right Hemisphere OR cerebral ischemia-reperfusion injury OR cerebral ischemia reperfusion injury) |
| --- | --- |
| **EMBASE** | 1 Untranslated RNA/  2 npcRNA/  3 RNA, Nontranslated/  4 Nontranslated RNA/  5 RNA, Non-Peptide-Coding/  6 Non-Peptide-Coding RNA/  7 RNA, Non Peptide Coding/  8 RNA, Non-Protein-Coding/  9 Non-Protein-Coding RNA/  10 RNA, Non Protein Coding/  11 RNA, Noncoding/  12 Noncoding RNA/  13 RNA, Non-Coding/  14 Non-Coding RNA/  15 RNA, Non Coding/  16 1-15/or  17 Noncoding RNA, Long/  18 lncRNA/  19 Long ncRNA/  20 ncRNA, Long/  21 RNA, Long Non-Translated/  22 Long Non-Translated RNA/  23 Non-Translated RNA, Long/  24 RNA, Long Non Translated/  25 Long Non-Coding RNA/  26 Long Non Coding RNA/  27 Non-Coding RNA, Long/  28 RNA, Long Non-Coding/  29 Long Non-Protein-Coding RNA/  30 Long Non Protein Coding RNA/  31 Non-Protein-Coding RNA, Long/  32 RNA, Long Non-Protein-Coding/  33 Long Noncoding RNA/  34 RNA, Long Untranslated/  35 Long Untranslated RNA/  36 Untranslated RNA, Long/  37 Long ncRNAs/  38 ncRNAs, Long/  39 Long Intergenic Non-Protein Coding RNA/  40 Long Intergenic Non Protein Coding RNA/  41 LincRNAs/  42 LINC RNA/  43 LincRNA/  44 17-43/or  45 MicroRNA/  46 miRNAs/  47 Micro RNA/  48 RNA, Micro/  49 miRNA/  50 Primary MicroRNA/  51 MicroRNA, Primary/  52 Primary miRNA/  53 miRNA, Primary/  54 pri-miRNA/  55 pri miRNA/  56 RNA, Small Temporal/  57 Temporal RNA, Small/  58 stRNA/  59 Small Temporal RNA/  60 pre-miRNA/  61 pre miRNA/  62 45-61/or  63 circRNAs/  64 Closed Circular RNA/  65 Circular RNA, Closed/  66 RNA, Closed Circular/  67 Circular RNA/  68 Circular RNAs/  69 RNAs, Circular/  70 circRNA/  71 Circular Intronic RNA/  72 Intronic RNA, Circular/  73 RNA, Circular Intronic/  74 ciRNA/  75 63-74/or  76 16 or 44 or 62 or 75  77 Cerebral Infarctions/  78 Infarctions, Cerebral/  79 Infarction, Cerebral/  80 Cerebral Infarct/  81 Cerebral Infarcts/  82 Infarct, Cerebral/  83 Infarcts, Cerebral/  84 Cerebral Infarction, Left Hemisphere/  85 Left Hemisphere, Infarction, Cerebral/  86 Infarction, Left Hemisphere, Cerebral/  87 Left Hemisphere, Cerebral Infarction/  88 Cerebral, Left Hemisphere, Infarction/  89 Infarction, Cerebral, Left Hemisphere/  90 Subcortical Infarction/  91 Infarction, Subcortical/  92 Infarctions, Subcortical/  93 Subcortical Infarctions/  94 Posterior Choroidal Artery Infarction/  95 Anterior Choroidal Artery Infarction/  96 Cerebral Infarction, Right Hemisphere/  97 Right Hemisphere, Cerebral Infarction/  98 Infarction, Right Hemisphere, Cerebral/  99 Right Hemisphere, Infarction, Cerebral/  100 Cerebral, Right Hemisphere, Infarction/  101 Infarction, Cerebral, Right Hemisphere/  102 cerebral ischemia-reperfusion injury/  103 cerebral ischemia reperfusion injury/  104 77-103/or  105 76 and 104 |
